# Systematic discovery of neoepitope–HLA pairs for neoantigens shared among patients and tumor types

**DOI:** 10.1038/s41587-023-01945-y

**Published:** 2023-10-19

**Authors:** Hem R. Gurung, Amy J. Heidersbach, Martine Darwish, Pamela Pui Fung Chan, Jenny Li, Maureen Beresini, Oliver A. Zill, Andrew Wallace, Ann-Jay Tong, Dan Hascall, Eric Torres, Andy Chang, Kenny ‘Hei-Wai’ Lou, Yassan Abdolazimi, Christian Hammer, Ana Xavier-Magalhães, Ana Marcu, Samir Vaidya, Daniel D. Le, Ilseyar Akhmetzyanova, Soyoung A. Oh, Amanda J. Moore, Uzodinma N. Uche, Melanie B. Laur, Richard J. Notturno, Peter J. R. Ebert, Craig Blanchette, Benjamin Haley, Christopher M. Rose

**Affiliations:** 1 Genentech, South San Francisco, CA USA; 2 Adaptive Biotechnologies, Seattle, WA USA

**Keywords:** Targeted therapies, Mass spectrometry, Target identification

## Abstract

The broad application of precision cancer immunotherapies is limited by the number of validated neoepitopes that are common among patients or tumor types. To expand the known repertoire of shared neoantigen–human leukocyte antigen (HLA) complexes, we developed a high-throughput platform that coupled an in vitro peptide–HLA binding assay with engineered cellular models expressing individual HLA alleles in combination with a concatenated transgene harboring 47 common cancer neoantigens. From more than 24,000 possible neoepitope–HLA combinations, biochemical and computational assessment yielded 844 unique candidates, of which 86 were verified after immunoprecipitation mass spectrometry analyses of engineered, monoallelic cell lines. To evaluate the potential for immunogenicity, we identified T cell receptors that recognized select neoepitope–HLA pairs and elicited a response after introduction into human T cells. These cellular systems and our data on therapeutically relevant neoepitopes in their HLA contexts will aid researchers studying antigen processing as well as neoepitope targeting therapies.

## Abstract (web summary)

A large resource of shared tumor neoepitopes aims to accelerate cancer immunotherapy.

## Main

T cells play a critical role in eliminating cancer cells^1,2^, and immunotherapies that enhance endogenous tumor-specific T cell activity (for example, cancer vaccines) or introduce T cells that target neoantigens have shown clinical efficacy^3,4^. Several neoantigen-directed therapies require the presentation of neoepitopes—8–11 amino acid peptides derived from mutated proteins—by polymorphic human leukocyte antigen class I (HLA-I, hereafter HLA) molecules on the surface of tumor cells. T cell receptors (TCRs) interact with a cognate peptide in the context of an HLA complex such that the therapeutic target comprises both the neoepitope sequence and the HLA subtype. Despite promise in the clinic, the application of neoantigen-specific therapeutics is limited, at least in part, by the scope of verified neoepitope–HLA combinations, particularly those that may be common across tumor subtypes or patient populations.

Precision T cell therapies target two broad categories of neoantigens: private and shared^5^. Private neoantigens are somatic variants unique to an individual’s tumor and represent the majority of mutations that arise during cancer progression^1^. Shared neoantigens recur across many patients due to common oncogenic mutations in proteins such as KRAS, EGFR, TP53 and BRAF^2,3^. Prior knowledge of shared neoantigens could enable prioritization of target epitopes and a path toward off-the-shelf therapeutics for patients with an appropriate tumor mutation and HLA haplotype.

Discovery of shared neoepitopes presented in their native context is challenging due to the tremendous number of possible neoepitope–HLA combinations. For each coding variant, there are 38 possible 8–11 amino acid epitopes with the potential to bind to thousands of distinct HLA alleles depending on the amino acid composition of the neoepitope. If considering only 15 HLA alleles and 50 shared cancer neoantigens, more than 28,000 neoepitope–HLA pairs could be formed. Although progress has been made toward the development of computational methods to predict neoepitope–HLA binding events^4^, they are not yet fully able to identify which peptides are processed and presented in a cellular context.

We present here a scalable pipeline for neoepitope–HLA pair discovery. For this, we selected 47 cancer mutations and 15 prevalent HLA alleles to define the neoepitope landscape and, by extension, putative clinical targets. We then employed a high-throughput HLA binding assay^5,6^ and NetMHCpan-4.0 (ref. ^7^) to experimentally and computationally identify neoepitope–HLA combinations for follow-up. Neoepitope–HLA pairs observed through both methods were assayed for presentation using untargeted and targeted mass spectrometry (MS) analyses of HLA monoallelic cell lines modified to express ~25 amino acid segments corresponding to each of the 47 cancer neoantigens, resulting in 86 observed neoepitope–HLA pairs. To assess the therapeutic potential for these targets, we used TCRs discovered through Multiplex Identification of T cell Receptor Antigen (MIRA) (Adaptive Biotechnologies)^8^ assays and demonstrated mutant-selective T cell targeting of cells expressing these neoepitopes.

## Results

### Clinico-genomics analysis of shared cancer neoantigens

**Fig. 1:**
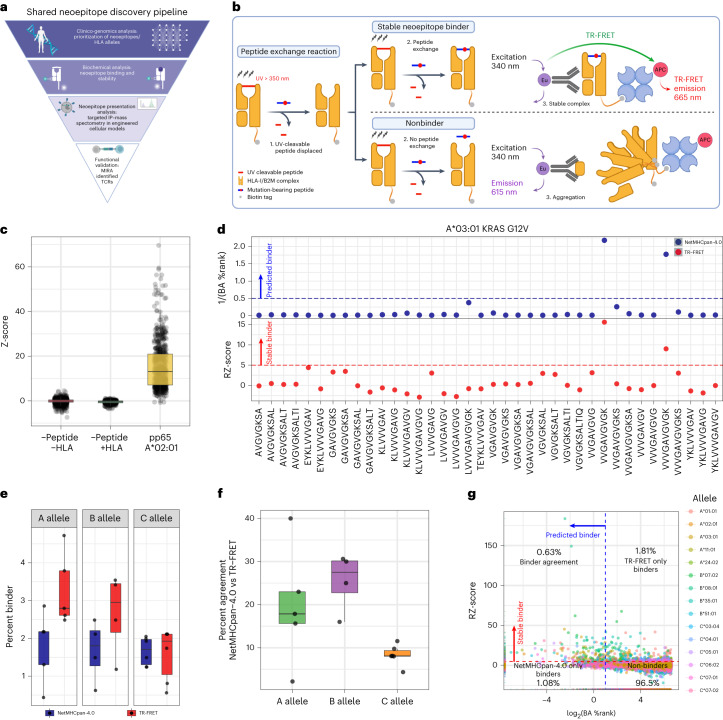
A shared neoepitope discovery pipeline featuring characterization of neoepitope–HLA binding through a high-throughput TR-FRET assay and NetMHCpan-4.0 prediction. **a**, Overview of the shared neoepitope discovery pipeline. **b**, Schematic diagram of the TR-FRET assay used to measure stable neoepitope–HLA binding. In brief, HLA monomers bound to UV-cleavable peptides are exposed to UV light in the presence of mutation-bearing candidate neoepitopes. Successful exchange of the candidate peptide will lead to complex stabilization and TR-FRET emission (top). Unsuccessful exchange will lead to aggregation and no TR-FRET emission (bottom). **c**, TR-FRET data for all controls measured within the screen. Z-score was calculated as compared to −Peptide/+HLA controls for each 384-well plate. The box represents the interquartile range; the line represents the median value; and the whiskers represent the minimum and maximum values (excluding outliers). Each dot represents an individual well measurement (−Pep,−HLA *n* = 1,477; −Pep,+HLA *n* = 368; pp65,+A*02:01 *n* = 623). **d**, Representative TR-FRET results for KRAS G12V/A*03:01 comparing NetMHC BA percentile rank (%Rank, blue) and RZ-score (red). **e**, Percent of neoepitope–HLA combinations that were determined to be stable binders by TR-FRET (red) or NetMHC (blue) across the HLA A, B and C alleles. The box represents the interquartile range; the line represents the median value; and the whiskers represent the minimum and maximum values (excluding outliers). Each dot represents the count of binders for a single allele (A allele,NetMHC *n* = 5; A allele,TR-FRET *n* = 5; B allele,NetMHC *n* = 4; B allele,TR-FRET *n* = 4; C allele,NetMHC *n* = 6; C allele,TR-FRET *n* = 6). **f**, Percent of neoepitope–HLA pairs found to be binders by NetMHC and/or TR-FRET across the A (green), B (purple) and C (orange) alleles, with each dot representing a single allele. The box represents the interquartile range; the line represents the median value; and the whiskers represent the minimum and maximum values (excluding outliers). Each dot represents the percent agreement for each allele (A allele *n* = 5, B allele *n* = 4, C allele *n* = 6). **g**, Scatter plot of TR-FRET RZ-score and NetMHC BA %Rank. The dashed red line represents the cutoff for stable binders as measured by TR-FRET, where values higher than the red line are considered a stable binder. The dashed blue line represents the cutoff for binders based on NetMHC analysis, where values lower than the blue line are considered binders. **a** and **b** were created with BioRender.com.

To establish a computational and experimental pipeline for neoepitope–HLA discovery, we first identified the most common recurrent point mutations across cancer types within a compendium of sequencing data from tumor and normal tissue samples^9^, filtering at a per-indication case prevalence of 2% (Fig. 1a). This led to a list of 36 shared cancer neoantigens (Supplementary Table 1). Next, we mined the Allele Frequency Net Database (AFND) and The Cancer Genome Atlas (TCGA) to catalog common haplotypes, narrowing to those with a carrier frequency of at least 10% in TCGA and an allele frequency of at least 5% in AFND. This analysis led to a list of 16 HLA alleles that combined with the 36 selected neoantigens to provide the foundation for development of our platform (Supplementary Table 1).

### High-throughput TR-FRET analysis of neoepitope–HLA stability

To survey all potential neoepitopes between candidate cancer neoantigens and selected HLA alleles, a high-throughput time-resolved fluorescence energy transfer (TR-FRET) assay based on peptide-mediated stabilization of conditional HLA complexes was developed (Fig. 1b)^5^. Our neoantigen target set consisted of 36 shared cancer neoantigens identified above along with 11 additional tumor antigens. Separately, 15 of 16 prioritized HLA variants were viable in the conditional HLA complex format (Fig. 1b). Together, this permitted the characterization of 24,149 neoepitope–HLA complexes after eliminating overlapping cancer neoepitopes as well as one allele due to synthesis challenges (Supplementary Table 2).

Conditional HLA complexes, pre-loaded with ultraviolet (UV)-cleavable peptides, were incubated with a neoepitope of interest at 100-fold molar excess and exposed to UV light for 25 min. This reaction leads to conditional ligand cleavage and conversion of the peptide from a stable high-affinity ‘binder’ to an unstable binder that dissociates from the HLA groove. In the presence of a binding neoepitope, peptide exchange would stabilize the HLA complex, whereas a lack of binding results in complex dissociation. Complex stability was monitored using fluorescence of a TR-FRET donor (europium) conjugated to an anti-β2M antibody and a TR-FRET acceptor conjugated to streptavidin, which bound to the biotin tag on the HLA alpha chain, where a TR-FRET signal would be observed only if the complex remained intact. TR-FRET signals were quantified based on the ratio of relative fluorescent units (RFUs), and signals were subjected to a double normalization to generate a robust Z-score (RZ-score). Any neoepitope–HLA combination with an RZ-score ≥5 was considered a ‘stable binder’, which was a conservative measure based on prior assessment with our positive control CMV-peptide/HLA-A*02:01 complex. A Z-score ≥5 captured 90% of positive control binding events without identifying false-positive binders (Fig. 1c).

NetMHCpan-4.0 (hereafter NetMHC) was employed to better understand how our TR-FRET results compared to computational prediction methods^10,11^. We considered NetMHC binding affinity (BA) percentile rank (%Rank) relative to TR-FRET results and eluted ligand (EL) %Rank to determine if a neoepitope was predicted to be presented (%Rank ≤2). Representative data for KRAS G12V peptides binding to A*03:01 showed two previously described neoepitopes^12,13^, VVGAVGVGK and VVVGAVGVGK, as binders with both approaches (Fig. 1d). Further examination of neoantigen–HLA combinations revealed variable concordances between the TR-FRET and NetMHC results (Supplementary Fig. 1a,b). Assessment of KRAS G12D peptides with C*08:02 found a known neoepitope (GADGVGKSAL)^14^ to be a binder by both methods (Supplementary Fig. 1c).

When measured as a percentage of all potential neoepitope–HLA complexes, TR-FRET generally identified more stable binders as compared to NetMHC (Fig. 1e and Supplementary Fig. 1d). We found that the percent agreement between NetMHC prediction and TR-FRET when classifying binders was generally less than 30% (Fig. 1f and Supplementary Fig 1e), whereas much stronger agreement was found for non-binders only (Supplementary Fig. 1f,g). For further comparison, TR-FRET RZ-scores and NetMHC %Ranks were plotted for all candidate neoepitope–HLA pairs, demonstrating that 0.63% of neoepitope–HLA pairs were probable binders by both methods (Fig. 1g and Supplementary Fig. 2a). The different methods identified a similar percentage of additional binding events for neoepitope–HLA pairs, demonstrating that each has the potential to uncover unique binding combinations (Fig. 1g and Supplementary Fig. 2b). These findings highlight the power of our high-throughput TR-FRET assay to identify an expanded and complementary set of neoepitope–HLA pairs relative to computational prediction and suggest that co-deployment of both approaches would be needed for comprehensive neoepitope discovery.

### Generation of monoallelic cells co-expressing 47 neoantigens

Despite observed peptide–HLA stabilization in vitro or computational prediction of an interaction, mutant protein expression and processing may not result in neoepitope presentation in a cellular context^15–17^. For this reason, candidate neoepitope validation typically requires evidence of direct physical association with surface-bound HLA via HLA immunoprecipitation (HLA-IP) followed by MS. This process has been enhanced through the use of engineered ‘HLA monoallelic’ cell lines, although these have largely relied on endogenous mutant protein expression or expression of relatively few mutant transgenes, thus limiting throughput^13,18,19^.

**Fig. 2:**
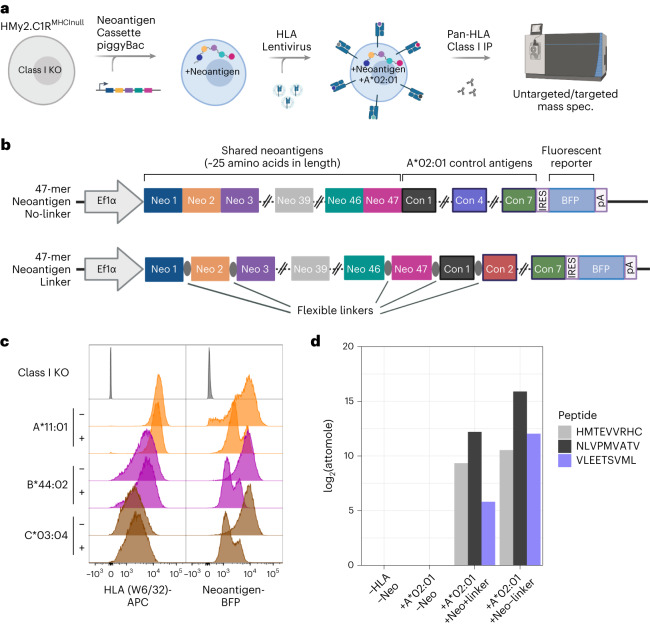
Generation of HLA class I monoallelic cell lines that stably express a polyantigen cassette containing 47 shared cancer neoantigens. **a**, Process overview for the cell engineering and MS analysis of peptides presented by polyantigen-expressing HLA monoallelic cell lines. KO, knockout. **b**, Vector map of the piggyBac polyantigen expression constructs used in this study. A single transcript containing 47 tandem neoantigens followed by seven control peptides and an IRES-linked mTagBFP2 (BFP) reporter is driven by an EF1a promoter. Neoantigens were either directly concatenated (no-linker) or interspersed by short flexible linker sequences (linker). **c**, Flow cytometric detection of HLA expression (W6/32 antibody-APC) and polyantigen cassette reporter (BFP) in selected cell lines or HLA knockout parental line. Here, ‘−’ indicates the absence of linkers and ‘+’ indicates presence of linkers in the polyantigen construct. **d**, Targeted immunopeptidomic detection of a previously described A*02:01-presented TP53 neoepitope (HMTEVVRHC, position 39) as well as two neoantigen control peptides from pp65 (NLVPMVATV, control 1) and IE-1 (VLEETSVML, control 4) known to be presented by the A*02:01 allele. **a** and **b** were created with BioRender.com.

We anticipated that co-expression of all 47 candidate neoantigen sequences (concatenated ~25 amino acid segments centered on the mutated position) within a single HLA-null cell line would improve throughput of monoallelic cell line generation and subsequent validation of TR-FRET/NetMHC-identified neoepitope–HLA pairs by targeted MS (Fig. 2a). For this, we selected the HMy2.C1R (C1R) lymphoblast cell line, which lacks HLA-A and HLA-B^20,21^. To generate a full C1R^HLAnull^ cell line, the HLA-C allele (HLA-C*04:01) was disrupted using CRISPR–Cas9, and the HLA-null population was enriched by fluorescence activated cell sorting (FACS) (Supplementary Fig. 3a–c).

Local amino acid sequence context may affect antigen processing^22^. Accordingly, we engineered unique C1R^HLAnull^ lines to stably express concatemers of all 47 prioritized neoantigens that were separated, or not, by short, flexible amino acid linkers (Fig. 2b). Subsequent introduction of the 15 HLA alleles as individual transgenes through stable lentiviral transduction of the linker and no-linker neoantigen-expressing C1R^HLAnull^ cell lines resulted in 30 total cell populations (Fig. 2c and Supplementary Fig. 3d,e). To validate functionality of the polyantigen cassettes, the linker and no-linker neoantigen constructs contained an identical set of seven known HLA-A*02:01-presented epitopes. HLA-IP followed by targeted MS analysis confirmed presentation of two control peptides and a previously described TP53 R175H^23^ neoepitope in both the linker and no-linker HLA-A*02:01-engineered cells (Fig. 2d).

### Detection of neoepitopes presented on engineered monoallelic cells

Both targeted and untargeted MS were applied for neoepitope discovery across the panel of monoallelic cell lines. Untargeted MS analysis enabled unbiased identification of peptides from the entire immunopeptidome, whereas targeted analysis facilitated detection of peptides presented at low copies per cell but was constrained to prioritized sequences from our TR-FRET/NetMHC analyses.

**Fig. 3:**
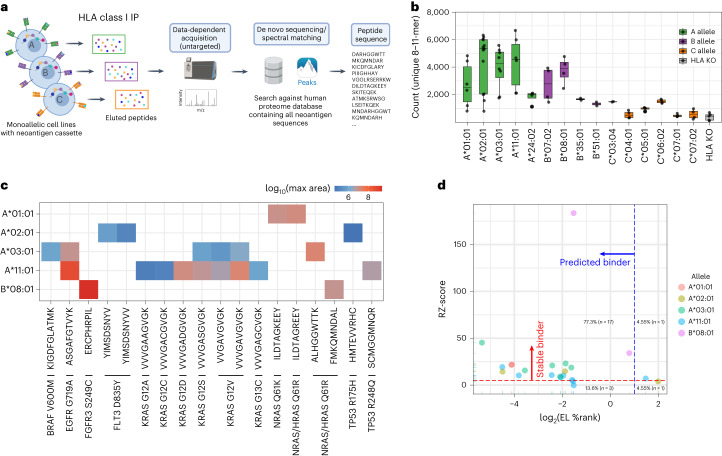
Untargeted immunopeptidomic analysis of monoallelic cell lines expressing the polyantigen cassette. **a**, Workflow for untargeted immunopeptidomic analysis of monoallelic cell lines containing the polyantigen cassette. **b**, Number of unique 8–11-mer peptides identified in untargeted immunopeptidomic analysis. The box represents the interquartile range; the line represents the median value; and the whiskers represent the minimum and maximum values (excluding outliers). Each dot represents a separate analysis beginning with a replicate cell pellet (A*01:01 *n* = 6, A*02:01 *n* = 13, A*03:01 *n* = 8, A*11:01 *n* = 6, A*24:02 *n* = 4, B*07:02 *n* = 4, B*08:01 *n* = 4, B*35:01 *n* = 2, B*51:01 *n* = 2, C*03:04 *n* = 2, C*04:01 *n* = 4, C*05:01 *n* = 4, C*06:02 *n* = 4, C*07:01 *n* = 4, C*07:02 *n* = 4, HLAKO *n* = 5). **c**, Identified shared cancer neoantigen epitopes. The color scale represents the log_10_ largest area across all analyses. **d**, Comparison of TR-FRET RZ-score and NetMHC EL percentile rank (%Rank) score for each epitope identified through untargeted immunopeptidome analysis. **a** was created with BioRender.com.

Untargeted MS analysis was performed with a semi-automated workflow resulting in 218–6,663 unique 8–11-mer peptides identified from each cell population (Fig. 3a,b). The number of 8–11-mer peptides and general sequence features for each allele overlapped regardless of the polyantigen linker status (Supplementary Fig. 4a) and confirmed that presented peptides fit known motifs (Supplementary Fig. 5). Expression of the polyantigen cassette was confirmed by detection of control viral epitopes from A*02:01 and A*11:01 monoallelic cells (Supplementary Fig. 4b) as well as epitopes from an integrated blue fluorescence protein (BFP) selection marker across eight different HLA alleles (Supplementary Fig. 4c).

From our untargeted analyses, we observed 22 neoepitope–HLA pairs and several peptides from non-mutation-bearing regions of the polyantigens. Neoepitopes corresponded to 15 shared neoantigens across five HLAs, representing ~5.4% of neoepitope–HLA pairs predicted by NetMHC and ~3.7% of neoepitope–HLA pairs identified within the TR-FRET assay (Fig. 3c and Supplementary Table 3). Of the 22 neoepitope–HLA pairs, 10 were previously described in the literature, and the remaining 12 were thought to be novel based on a search of Tantigen^24^, CAatlas^25^ and NEPdb^26^ and an extended literature survey (Supplementary Table 3). TR-FRET and NetMHC showed excellent concordance for all 22 identified pairs; 17 were identified as binders by both approaches (Fig. 3d). One and three neoepitope–HLA pairs were uniquely identified as hits by TR-FRET and NetMHC, respectively, demonstrating that each approach can predict distinct neoepitope subsets (Fig. 3d). One neoepitope–HLA pair (TP53 R175H (HMTEVVRHC)/A*02:01) represented an exception. This pair had a TR-FRET RZ-score of 3.9 and a NetMHC EL %Rank of 3.98 and was not considered a hit by either approach, demonstrating that false negatives remain possible.

**Fig. 4:**
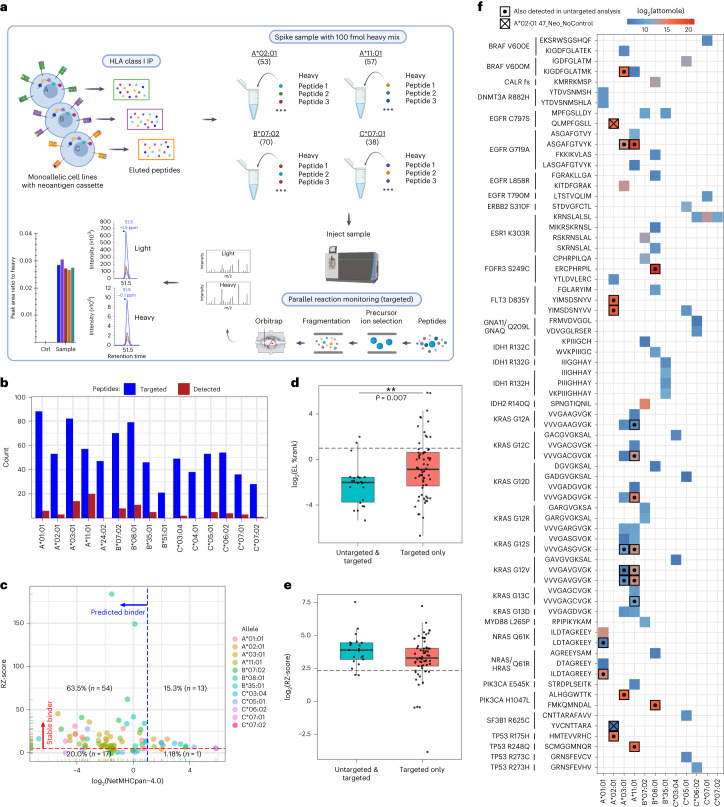
Targeted immunopeptidomic analysis of monoallelic cell lines expressing a polyantigen cassette. **a**, Targeted immunopeptidomic workflow for the analysis of candidate neoepitopes within monoallelic cell lines expressing a polyantigen cassette. **b**, The number of targeted (blue) and detected (red) shared cancer neoantigen epitopes within each targeted assay. **c**, Comparison of TR-FRET RZ-score and NetMHC EL %Rank score for each epitope identified through targeted immunopeptidome analysis. **d**, NetMHC EL %Rank scores for neoepitopes detected in both untargeted and targeted (teal) analysis or targeted analysis alone (red). The box represents the interquartile range; the line represents the median value; and the whiskers represent the minimum and maximum values (excluding outliers). Each dot represents a neoepitope–HLA pair (untargeted & targeted *n* = 22, targeted only *n* = 64). The *P* value was calculated using a Wilcoxon test (two-sided). **e**, Same analysis as **d** but for TR-FRET RZ-scores. **f**, Summary of neoepitope–HLA pairs detected from shared cancer neoantigens. Color represents attomol of neoepitopes detected on column during analysis. Bolded squares with centered dots represent neoepitopes also detected in untargeted analysis. A square with an ‘X’ indicates A*02:01-specific neoepitopes that were detected in a cell line containing a polyantigen construct lacking control neoantigen sequences. **a** was created with BioRender.com.

Although we surmised that targeted MS analysis would improve detection of presented neoepitopes, this relied on heavy isotope-labeled standard peptides. As such, a logistically challenging synthesis of 1,786 peptides (47 neoantigens × 38 possible mutation-bearing candidate neoepitopes) would be needed to screen all potential neoepitopes from our monoallelic cell lines. Therefore, we used the TR-FRET results as a preliminary screen and synthesized all 397 peptides with an RZ-score ≥5. Due to the complementarity of TR-FRET and NetMHC results, an additional 81 peptides were synthesized that had an RZ-score <5 and NetMHC %Rank ≤2. The 479 peptides were divided into 15 HLA allele-specific pools comprising 21–88 peptides (Fig. 4a,b).

Targeted MS analysis identified 86 neoepitope–HLA pairs across 12 different alleles and 36 neoantigens, representing a ~4-fold improvement compared to untargeted MS analysis (Fig. 4b and Supplementary Table 3). After a search of the literature and relevant databases, we determined that 21 of the neoepitope–HLA pairs were described previously, and 65 were novel (Supplementary Table 3). Twenty of 86 neoepitope–HLA pairs identified across untargeted and targeted analyses were associated with A*11:01. This was likely due to the presence of eight distinct KRAS neoantigen sequences in the polyantigen cassette, as 14 of 20 A*11:01-specific and nine of 14 A*03:01-specific neoepitopes mapped to KRAS G12X or G13X neoantigens.

To assess the value of using TR-FRET and NetMHC results to select peptides for targeted MS, we plotted RZ-score versus NetMHC %Rank for each of the observed 86 neoepitope–HLA pairs (Fig. 4c). This revealed that 55 neoepitopes were stable binders by TR-FRET and predicted to be presented by NetMHC. Thirteen neoepitope–HLA pairs were found as hits in TR-FRET only, whereas 18 neoepitope–HLA pairs were hits in NetMHC alone (Fig. 4c). To understand the binding characteristics of neoepitope–HLA pairs identified by targeted analysis alone, we plotted RZ and NetMHC %Rank scores for peptides observed in both untargeted and targeted analysis compared to peptides found only in targeted analysis (Fig. 4d,e). Neoepitope–HLA pairs identified by targeted analysis alone had a broader range of NetMHC %Rank and TR-FRET RZ-scores relative to neoepitopes also detected in untargeted analysis (Fig. 4d,e). This suggests that targeted analysis can identify neoepitopes that are weaker binders compared to those observed by untargeted means.

Targeted MS permits absolute quantification of peptide presentation across neoepitopes. Overall, the measured amount of neoepitope presentation spanned from 60 amol to 2.5 pmol (Fig. 4f) and was consistent across independent replicates of cell line growth and sample preparation (Supplementary Fig. 6). Two peptides detected by untargeted MS, EGFR G719A (ASGAFGTVYK) and FGFR3 S249C (ERCPHRPIL), exhibited the highest absolute quantities (Fig. 4f). When the absolute amounts of neoepitopes detected were compared to RZ-score, NetMHC EL %Rank or NetMHC BA %Rank for each allele, no clear correlation could be found (Supplementary Fig. 7a–c). This suggests that each score has predictive value for neoantigen presentation but also that these cannot be used to estimate the absolute amount presented.

### Polyantigen cassette design impacts neoepitope presentation

The polyantigen sequence included neoantigens with known A*02:01-binding epitopes to confirm translation, processing and presentation of the cassette. It was possible that the controls could compete with experimental neoepitopes, thus creating an avenue for false negatives. To evaluate this, a separate A*02:01 cell line was created that stably expressed a no-linker polyantigen cassette lacking control sequences. Upon analysis of the no-control line, two additional neoepitopes were detected: YVCNTTARA (SF3B1 R625C; RZ-score = 16; EL %Rank = 5.3) and QLMPFGSLL (EGFR C797S; RZ-score = 7; EL %Rank = 0.21) (Fig. 4f, squares with ‘X’). These results suggest that strong binding peptides could inhibit presentation of certain neoepitopes, and a revised workflow may omit control sequences from the polyantigen cassette.

Polyantigen cassette length is an important consideration when designing cancer vaccines, and a concern that translation of neoantigens at the C-terminal/3′ end of the cassette will be decreased may have factored into the use of shorter cassettes in clinical settings (for example, 10-mer or 34-mer)^27^. To characterize the translation efficiency of our 47-mer polyantigen transgene, we performed ribosome profiling (Ribo-Seq) on A*02:01 monoallelic cells containing either the linker or no-linker cassette with A*02:01 controls (Supplementary Fig. 8a,b). These analyses demonstrated consistent translation across the no-linker polyantigen cassette, whereas the cassette containing linkers had a substantial decrease in translation after ~20 neoantigen sequences (Supplementary Fig. 8a,b).

We next sought to understand if the difference in translation between cassette designs was reflected within our targeted immunopeptidomics results. For this, we plotted the highest attomole abundance of presented peptide for each neoantigen (irrespective of HLA) versus neoantigen position within the linker and no-linker polyantigen cassettes (Supplementary Fig. 8c). This revealed a potential bias toward presentation of peptides derived from the first ~20 neoantigen sequences regardless of format. Within the portion of the polyantigen cassette that exhibits lower translation, we detected six additional neoepitope–HLA pairs from cells expressing the no-linker cassette, suggesting that the no-linker format may be advantageous for assaying ≥20 target sequences (Supplementary Fig. 8c).

For neoepitopes detected in both the linker and no-linker cell lines, there was not a clear difference in the maximum presentation, suggesting that positional effects detected in the Ribo-Seq data could be buffered at the level of presentation (Supplementary Fig. 8c). This was further supported by roughly equivalent presentation of KRAS G12X and G13X neoepitopes (which are identical except for the mutated residue) across the polyantigen cassette (Supplementary Fig. 8d). To evaluate the impact of linkers more broadly, we plotted the highest absolute amount of neoepitope presented and found that presentation of some neoepitopes increased in the presence of linkers while presentation of other neoepitopes decreased (Supplementary Figs. 9a,b and 10). Together, these data demonstrate that the no-linker polyantigen cassette enabled detection of a greater number of neoepitope–HLA pairs. However, if a neoepitope was detected in linker and no-linker cells, the presence of linkers did not impact abundance of presentation in a consistent manner.

### Validation of neoepitope presentation from full-length protein

**Fig. 5:**
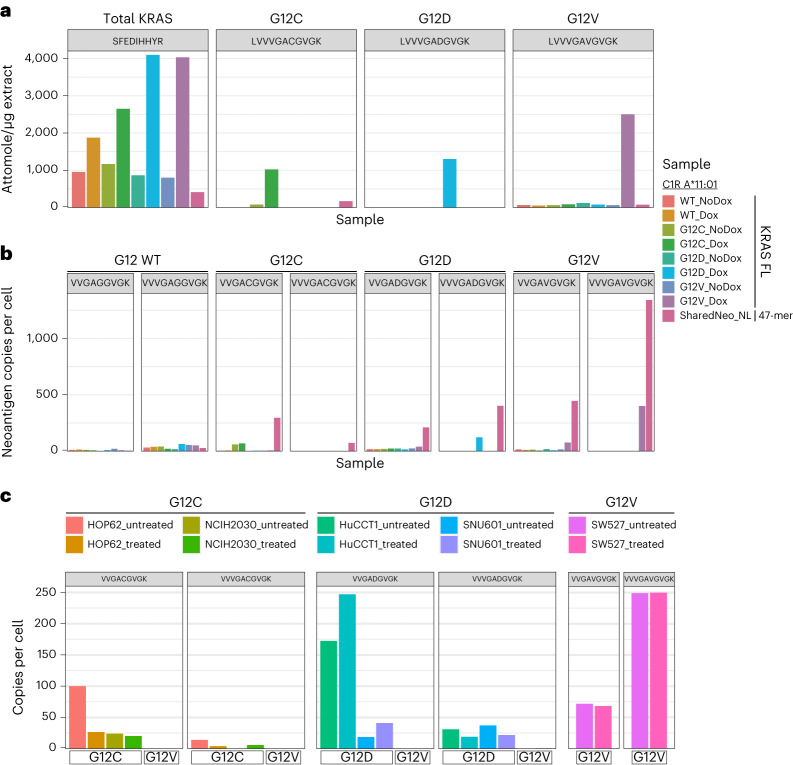
Presentation of KRAS neoepitopes derived from exogenous and endogenous expression of full-length mutant protein. **a**,**b**, A*11:01 monoallelic cells were engineered to express doxycycline (dox)-inducible full-length (FL) KRAS mutant proteins (G12C, G12D and G12V). These were compared against an A*11:01 monoallelic cell line containing the no-linker polyantigen cassette. **a**, Absolute amount of KRAS wild-type (WT) and mutant proteins in the cell lysate by targeted MS. **b**, Copies per cell of presented KRAS 9-mer (VVGAXGVGK) and 10-mer (VVVGAXGVGK) neoepitopes as measured by A*11:01 monomers containing heavy synthetic neoepitope peptides spiked in before affinity purification and targeted MS. **c**, Targeted immunopeptidomic analysis of neoepitopes in cell lines that endogenously express both KRAS and A*11:01. Two neoantigens for KRAS G12C (VVGACGVGK and VVVGACGVGK) and KRAS G12D (VVGADGVGK and VVVGADGVGK) were analyzed in cell lines that harbor KRAS G12C (HOP62 and NCIH2030), G12D (HuCCT1 and SNU601) or G12V (SW527). These neoepitopes are either novel or were described previously in non-endogenous systems. ‘Treated’ samples were treated with interferon-gamma.

Neoepitopes derived from a polyantigen construct may not reflect peptides processed from a full-length mutant protein. To address this, we developed four HLA-A*11:01 monoallelic C1R lines expressing an inducible, full-length wild-type, G12C, G12D or G12V mutant KRAS transgene and compared neoepitope presentation from these cell lines with a cell line expressing the same HLA and a no-linker polyantigen cassette. Expression of full-length variant proteins was confirmed using a whole-cell targeted proteomic assay comprising a peptide that can detect total KRAS as well as three unique peptides that measured individual KRAS mutants (Fig. 5a). Little to no mutant peptide signal was observed in total protein samples from the polyantigen cassette-expressing cell line (Fig. 5a).

We then performed HLA-IP and targeted MS to quantify presentation of previously identified 9-mer and 10-mer KRAS epitopes associated with HLA-A*11:01 (Fig. 5b)^12,13^. In cell lines expressing full-length mutant transgenes, clear induction of neoepitope presentation was observed for both G12V epitopes as well as the 10-mer epitope of G12D (Fig. 5b). From cells expressing the polyantigen cassette, all targeted mutant KRAS epitopes were detected and measured at higher absolute copies per cell compared to lines expressing full-length mutant proteins (Fig. 5b). Detection of KRAS peptides after HLA-IP but not from total cell protein suggested that the polyantigen concatemer was likely unstable and efficiently degraded, resulting in enhanced epitope presentation^28,29^. Therefore, monoallelic cells containing the polyantigen cassette provided a reliable, higher throughput and more sensitive system for discovery of neoepitopes from shared cancer neoantigens relative to cell lines expressing a full-length antigen.

Lastly, we sought to demonstrate that neoepitopes discovered by our pipeline can be identified within cells that endogenously co-express relevant proteins and HLA alleles. Targeted MS assays were used to quantify four neoepitopes—9-mer and 10-mer from KRAS G12C and G12D—within cell lines that express A*11:01 as well as KRAS G12C (HOP62 and NCIH2030), KRAS G12D (HuCCT1 and SNU601) or KRAS G12V (SW527) (Fig. 5c). One of these neoepitopes (KRAS G12C (VVGACGVGK)) has not previously been described, whereas the remaining three neoepitopes have been confirmed only within cellular systems that exogenously express the neoantigen^12^. We confirmed presentation of the four target neoepitopes within cell lines that harbor the target neoantigens (KRAS G12C and G12D), whereas there was no observed presentation in a control cell line that contained KRAS G12V (Fig. 5c). In both HOP62 and NCIH2030 cells, KRAS G12C 9-mer neoepitopes appeared to have higher absolute presentation as compared to the previously described 10-mer (Fig. 5c). Furthermore, whereas the presentation of the 10-mer KRAS G12D epitope was similar across HuCCT1 and SNU601 cells, presentation of the 9-mer KRAS G12D neoepitope was much higher within HuCCT1 (Fig. 5c). This suggests that presentation of slightly varying neoepitopes can differ substantially based on the cell line from which they are derived. In total, these data demonstrate that neoepitopes discovered through our pipeline can be found from both exogenously expressed full-length proteins and within systems that endogenously express both the HLA and neoantigen.

### Functional validation of tumor-specific antigen–HLA pairs

To determine whether neoepitopes identified through our workflow could be recognized by human T cells, we employed a modified multiplexed TCR discovery method^8^. Using two of the identified neoepitope–HLA pairs (FLT3-p.D835Y/A*02:01, PIK3CA-p.E545K/A*11:01) as examples, neoepitopes were first allocated to peptide pools in unique combinations before healthy human donor CD8^+^ T cells were expanded using autologous monocyte-derived dendritic cells, restimulated with the neoepitope peptide pools, sorted for activation marker upregulation and subjected to TCRβ sequencing. This method was used for donors spanning a range of HLA genotypes, enabling the association of TCRs with a variety of peptide–HLA pairs. However, owing to the multiallelic nature of donor cells, the HLA restriction of identified neoepitopes was not initially disambiguated among the 3–6 donor HLA alleles.

For neoepitopes that elicited a T cell response, associated TCRβ and TCRα sequences were determined using a parallel multiplexed assay^30^ that enabled construction of paired TCR expression vectors and the selection of candidate neoepitope-specific TCRs. The specificity and potential efficacy of each TCR were then assessed through cellular assays. TCR encoding in vitro transcribed mRNA was introduced via electroporation into primary human T cells, which were then incubated with either an increasing concentration of the candidate neoepitope in the presence of A*02:01^+^ T2 cells or monoallelic K562 cells that co-expressed an HLA allele and neoantigen of interest.

**Fig. 6:**
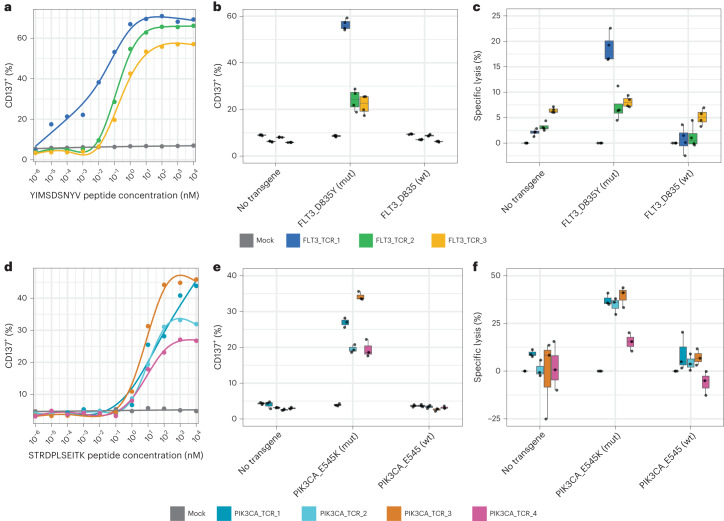
Discovery of neoepitope-specific TCRs demonstrates immunogenic potential of discovered neoepitope–HLA pairs. Human CD8^+^ T cells were transfected with either FLT3-p.D835Y-specific or PIK3CA-p.E545K-specific TCR RNA. **a**–**c**, CD137 expression was assessed after T cells transfected with FLT3-p.D835Y-specific TCRs were co-cultured overnight with YIMSDSNYV peptide-pulsed A*02:01^+^-T2 cells (**a**). CD137 expression (**b**) and specific lysis (**c**) were determined after co-culture with A*02:01^+^-K562 cells transfected with no RNA, transgene containing mutant FLT3-p.D835Y or transgene containing FLT3-D835 wild-type sequence. The box represents the interquartile range; the line represents the median value; and the whiskers represent the minimum and maximum values (excluding outliers) (*n* = 4). **d**–**f**, CD137 expression was assessed after T cells transfected with PIK3CA-p.E454K-specific TCRs were co-cultured overnight with STRDPLSEITK peptide-pulsed A*11:01^+^-K562 cells (**d**). CD137 expression (**e**) and specific lysis (**f**) were determined after co-culture with A*11:01^+^-K562 cells transfected with no RNA, transgene containing mutant PIK3CA-E545K or transgene containing PIK3CA-E545 wild-type sequence. The box represents the interquartile range; the line represents the median value; and the whiskers represent the minimum and maximum values (excluding outliers) (*n* = 3). mut, mutant; wt, wild-type.

We found dose-dependent upregulation of CD137 after 12-h co-culture of primary human CD8^+^ T cells transfected with predicted FLT3-p.D835Y/*02:01-specific TCRs in response to T2 cells incubated with exogenously delivered YIMSDSNYV peptide (Fig. 6a). Furthermore, these T cells were activated by and specifically killed monoallelic A*02:01-K562 cells expressing a mutant FLT3-p.D835Y transgene (minigene encoding 21 amino acids) but were not activated by and did not kill monoallelic A*02:01-K562 cells expressing a wild-type FLT3 transgene (Fig. 6b,c). These TCRs appear to be exquisitely specific for the mutant neoepitope, which is an important characteristic because a similar non-mutant epitope IMSDSNYVV was identified by untargeted analysis in A*02:01 monoallelic cells.

As a second proof of concept, T cells were transfected with predicted PIK3CA-p.E545K/HLA-A*11:01 TCRs and mixed with monoallelic A*11:01-expressing K562 cells incubated with an increasing concentration of the predicted neoepitope STRDPLSEITK (Fig. 6d). Here, TCR-transfected T cells demonstrated dose-dependent activation as measured by CD137 expression. Furthermore, these T cells demonstrated higher levels of activation and cell killing when mixed with A*11:01 K562 cells expressing a PIK3CA-p.E545K transgene (minigene encoding 21 amino acids) as compared to cells that expressed a wild-type PIK3CA transgene (Fig. 6e,f). Mutations that introduce anchor residues are thought to have high immunogenic potential because the immune system has not built tolerance to a similar wild-type epitope. For PIK3CA-p.E545K/A*11:01, the E → K mutation introduces an anchor residue within the context of A*11:01, and the wild-type STRDPLSEITE epitope was not detected in untargeted MS analyses of A*11:01 monoallelic cells. Although false negatives are anticipated in our MS workflow, the wild-type epitope was also not predicted to bind A*11:01 by NetMHC (12.8). Taken together, these data provide a clear mechanism for the specificity of PIK3CA-p.E545K TCRs for recognition of mutant PIK3CA as compared to wild-type and lend support for these TCRs as potential therapeutic candidates.

## Discussion

Most neoepitope discovery efforts have focused on a limited number of neoantigens and HLA alleles in the search for presented tumor-associated peptides^12,31,32^. We, therefore, developed a scalable, multiplexed platform that integrates a high-throughput binding assay, computational neoepitope binding prediction, complex cellular engineering of monoallelic cell lines and targeted MS to identify dozens of unique tumor-associated neoepitopes in context with specific HLA alleles, representing putative targets for neoantigen-based cancer immunotherapies.

In total, 24,149 potential neoepitope–HLA pairs were surveyed from 47 shared cancer neoantigens across 15 common HLA alleles, resulting in 844 stable combinations. From this, subsequent proteomic assessment using monoallelic cell lines identified 86 unique neoepitope–HLA pairs derived from 36 neoantigens across 12 HLA alleles. We selected two example combinations (FLT3-p.D835Y/A*02:01 and PIK3CA-p.E545K/A*11:01) for cell-based assays to validate a cohort of TCRs identified in a separate MIRA workflow, which demonstrated T cell activation or target cell killing and mutant peptide selectivity.

Despite a high rate of rediscovery for known peptide–HLA combinations with our platform, only a fraction of those found here were evaluated by HLA-IP-MS using cells that natively express the neoantigens and HLAs or express full-length mutant cDNAs. Also, our T cell/target cell co-culture assays relied on peptide pulsing or expression of the neoantigen from minigenes. A demonstration that T cells can be modified to target cells with endogenous expression of the newly observed neoepitope–HLA pairs would further substantiate our findings^13,23,33^. However, a paucity of appropriate cell lines poses a challenge to the study of endogenous neoepitope presentation, which may explain why only six of 21 previously reported neoepitope–HLA pairs described in the literature have been validated in a native context. This includes the KRAS G12V^13^, PIK3CA H104L^33^ and TP53 R175H^23^ neoepitope–HLA pairs validated in T cell targeting assays; neoepitope–HLA pairs from NRAS/HRAS Q61R^19^, NRAS Q61K^34^ and KRAS G12V^12,13,35^ that were detected through MS alone and a neoepitope–HLA pair from MYD88 L265P^36^ that was detected through ELISpot (Supplementary Table 3).

We extended this list by validating additional 9-mer and 10-mer neoepitope–HLA pairs from cells endogenously expressing either KRAS G12C or G12V and A*11:01 and found that copy/cell levels of neoepitope presentation as well as the relative ratios of 9-mer to 10-mer presentation varied across cell lines. This could have been due to differences in KRAS abundance in the cell and/or expression of genes involved in antigen processing, but a broader study of presentation across endogenous cell lines could reveal important insights into KRAS neoepitope presentation.

As described in previous studies, detection of neoepitopes by MS may be impacted by the amino acid composition of the peptides^37^. Thirty-four of 86 unique neoepitope–HLA pairs that we observed were associated with either A*03:01 or A*11:01. This was potentially due to the overrepresentation of KRAS variants in the polyantigen cassette but may also be due to a basic residue (lysine or arginine) at the C-terminus. Additional charges, either through additional basic residues or labeling with a chemical tag^34,38^, generally improve ionization, fragmentation and identification of peptides. The cysteine-containing HMTEVVRHC (TP53 R175H, A*02:01) was the only peptide that failed to reach significance by NetMHC or TR-FRET but was found by untargeted analysis. At least two details may explain this: cysteines have been underrepresented in MS data used to train prediction algorithms, and these residues can cause peptide dimerization in solution.

The datasets and tools that we developed represent a valuable and expandable resource for future studies of neoepitope presentation. For example, the TR-FRET dataset could be used for training or benchmarking neoepitope prediction algorithms that factor in neoepitope–HLA complex formation. Additionally, we provide raw data for untargeted and targeted MS analysis, enabling re-analysis with improved search algorithms^34^, peptide false discovery rate (FDR) determination^38^ or specific workflows that detect rare events within the antigen presentation pathway^39^. Monoallelic cell lines expressing the polyantigen cassette also represent a versatile system for characterizing the processing and presentation of private, shared and unconventional cancer antigens^39^.

The workflow that we describe provided insight into targets for future precision immunotherapies. In particular, few (86 total out of 24,149 initially screened neoepitope–HLA combinations (0.36%)) neoepitope–HLA pairs were detected as presented peptides. Neoepitopes for 14 of 36 cancer neoantigens were detected in the context of only one HLA allele, and, of the cancer neoantigens that presented epitopes across multiple alleles, nine were KRAS G12X or G13X mutations. Given this narrow spectrum of bona fide neoepitope–HLA targets, a broadened use of this platform and additional neoepitope–HLA discovery efforts will be needed to increase the coverage of patient populations most likely to benefit from shared neoantigen-specific immunotherapies.

## Methods

### Engineering of monoallelic polyantigen cassette-expressing HMy2.C1R cell lines

An HLA class-I null cell population was generated by CRISPR–Cas9-mediated gene disruption of the endogenous HLA-C locus in HMy2.C1R cells. Wild-type HMy2.C1R cells were electroporated with Cas9/RNP (Invitrogen) containing an HLA-C-specific sgRNA (Synthego, sequence: TTCATCGCAGTGGGCTACG) (Supplementary Fig. 3a) using an Amaxa V system (program D-023). After an expansion period, cells were stained with anti-pan-HLA (W6/32), and antigen-negative cells were enriched by FACS (Supplementary Fig. 3d,e). For flow cytometric data collection, experiments were performed on BD Celesta, BD Fortessa or BD Symphony machines using FACSDiva version8/version9 acquisition software.

The HLA null HMy2.C1R cells were stably engineered with a piggyBac neoantigen expression plasmid system designed to co-express 47 shared cancer neoantigens and seven A*02:01 control antigens. In brief, neoantigen segments (~25 amino acids each) were concatenated and converted to codon-optimized DNA segments (Integrated DNA Technologies) with or without a flexible linker separating most neoantigen sequences. A version of the neoantigen cassette without linkers and lacking control antigens was also generated for use in the A*02:01 monoallelic context. The polyantigen cassettes were synthesized and cloned into a piggyBac transposon plasmid downstream of a constitutive human EF1a and transcriptionally linked to an IRES-TagBFP2 reporter element. A separate hPGK promoter-driven puromycin resistance gene was included on the same vector for selection purposes. The polyantigen expression plasmid was co-electroporated with pBO (piggyBac transposase, Hera BioLabs) using a NEON system (Invitrogen) and a 100-µl kit (buffer R, 1,230 V, 20 ms, three pulses). Polyantigen-expressing cells were selected by culture in 1 µg ml^−1^ puromycin (Gibco) and further purified by FACS enrichment of the TagBFP2-positive population.

Unique HLA allele open reading frames (ORFs), each with a distinct 19-base pair (bp) DNA barcode, were cloned downstream of the human EF1a promoter (GenScript) in a custom-modified pLenti6.3 backbone (Thermo Fisher Scientific). Lentivirus was generated by Lipofectamine 2000 (Invitrogen)-mediated co-transfection of HEK293T cells with individual lenti-HLA expression constructs and packaging plasmids. Seventy-two hours after transfection, viral supernatant was harvested, filtered through a 0.45-µm filter and concentrated by LentiX concentrator reagent (Takara) following the manufacturerʼs recommended protocol. Linker or no-linker polyantigen-expressing HLA-null HMy2.C1R cells were transduced with HLA expression vectors via spin infection (800*g* for 30 min at room temperature with 8 µg ml^−1^ polybrene). Transgenic HLA-expressing cells subsequently were purified by magnetic bead-based enrichment (biotin-W6/32, BioLegend, SA-MACS). HLA allele identification was confirmed by barcode sequencing (amplicon primers: Fwd-TCCCAGAGCCACCGTTACAC, Rev-GACTTAACGCGTCCTGGTTGC; sequencing primer: CTGGTTGCAGGCGTTTAGCGT), and uniform expression of both the HLA allele and polyantigen cassette was confirmed by flow cytometry (Fig. 3c and Supplementary Fig. 2c,d) before analysis by MS.

For studies evaluating neoantigen presentation in the context of full-length neoantigen-containing proteins, A*11:01 monoallelic cell lines were stably engineered with a doxycycline (dox)-inducible piggyBac vector expressing wild-type or mutant alleles (G12D, G12C and G12V) of human KRAS. The KRAS allele of interest and an IRES-linked mCherry reporter were driven by a dox-responsive TRE3G promoter. A puromycin resistance gene and the Tet-on3G element were encoded on the same vector downstream of a constitutive hPGK promoter. After puro selection and expansion, KRAS expression was induced by treating cells with 1 µg ml^−1^ dox for 5 d before subsequent analysis.

### Clinico-genomics analysis

Prevalence data for common cancer mutations (single-nucleotide variants (SNVs) and insertions and deletions (indels)) were obtained from the Cancer Hotspots database (http://cancerhotspots.org)^6^ and cross-referenced with TCGA data obtained from the cBioPortal for Cancer Genomics (http://cbioportal.org). Prevalence data for common HLA alleles were obtained by tabulating HLA types from the AFND (http://allelefrequencies.net) and from TCGA normal samples. Allele frequency data for the HLA-A, HLA-B and HLA-C genes across seven selected populations were downloaded from the AFND in May 2020 (Supplementary Table 2). We focused on large datasets (*n* > 24,000 for each population) from the National Marrow Donor Program. HLA alleles with allele frequency below 1% in all populations were removed. We then calculated the overall allele frequency for each allele as the mean across all populations and used this overall frequency in filtering and ranking alleles. We also analyzed HLA typing data from TCGA that were generated by running PolySolver^40^ on the whole-exome data from 9,741 matched normal samples (Amir Horowitz, Icahn School of Medicine at Mount Sinai; Supplementary Table 1). We tabulated and ranked the most prevalent HLA alleles in TCGA and overlapped them with the list of prevalent alleles in the AFND, which allowed us to confirm that the HLA alleles we selected were generally present with similar frequencies in cancer and non-cancer settings.

From these datasets, the 47 most common cancer mutations were determined based on prevalence per cancer type; the 47 most common HLA alleles were also determined. Additional ranking of these mutations was performed that considered the overall prevalence of each cancer type and whether a neoantigen-specific therapy could be readily developed in a clinical setting.

### Predicted neoepitope landscape analysis

After translating mutations to peptide sequences, neoepitope–HLA binding predictions were generated using NetMHCpan-4.0 (ref. ^4^) on all combinations of 8-mer, 9-mer, 10-mer and 11-mer peptides derived from the 47 cancer neoantigens combined with 15 prevalent HLA alleles. Both BA and EL predictions were obtained, which were then used for downstream analysis. Predicted neoepitopes were defined as neoepitope–HLA combinations with mutant EL percentile rank <2.

### Automated high-throughput neoepitope exchange

See Supplementary Methods for protein expression, peptide synthesis and HLA–peptide refolding information. Peptides were diluted to 10 µM in 25 mM Tris pH 8.0, 150 mM NaCl, 4 mM EDTA and 4.35% ethylene glycol in 96-deep-well plates (VWR) using a Biomek i5 automated liquid handler (Beckman Coulter). The peptide–buffer mixtures were dispensed and reformatted into 384-well plates (Labcyte) at a volume of 47.5 µl per well, resulting in identical plates of up to 352 unique neoepitopes for screening against each of the 15 HLA alleles. The first two columns of the plate were reserved for controls. A*02:01 with and without exchange peptide was included on each plate as positive and negative controls for exchange, respectively. The well-characterized A*02:01-specific viral epitope, CMV pp65 peptide (NLVPMVATV, Elim Biopharmaceuticals), was plated in quadruplicate as a positive control for peptide exchange. Negative controls for exchange included wells to which no peptide was added and, instead, received ethylene glycol only during the peptide dilution step. Negative control wells for the HLA allele being screened were plated in octuplicate.

Using a MANTIS Liquid Handler (Formulatrix), 2.5 µl of 0.1 mg ml^−1^ UV peptide–HLA complexes was added to each well, with one HLA allele screened for binding per plate. Positive control wells received A*02:01, and negative control wells received either HLA A*02:01 or the HLA allele specific to the plate. The resultant peptide exchange reaction mixtures contained 10 µM peptide, 0.1 µM UV–HLA complex and 5% ethylene glycol (v/v).

The peptide exchange protocol was adapted from a previously described method^2^ by decreasing the UV exposure time and adding an incubation step after UV exposure. Plates containing the peptide exchange reaction mixtures were incubated under UV lamps (UVP 3UV Lamp, Analytik Jena) for 25 min using one lamp per plate. Plates were then sealed and incubated for 18 h at 37 °C.

### TR-FRET assay

The homogenous TR-FRET assay was carried out in MAKO 1,536-well white solid-bottom plates (Aurora Microplates). The total assay volume was 4 µl per well, including 2 µl of diluted samples and 2 µl of reagent mix. In brief, 1.8 µl per well of assay diluent (PBS, 0.5% BSA + 0.05% Tween 20 + 10 ppm proclin, Genentech) was added to the 1,536-well destination plate by a Multidrop Combi nL dispenser (Thermo Fisher Scientific). Then, 200 nl of 5 µg ml^−1^ HLAI complex sample was dispensed from the Echo-qualified 384-well source plate (Beckman Coulter) into the destination plate by an Echo 550 acoustic liquid dispenser (Beckman Coulter). After centrifugation for 3 min, 2 µl of master mix donor at 2 nM (europium mouse anti-human β2-microglobulin (β2M), BioLegend, custom labeled by PerkinElmer) and acceptor at 40 nM (SureLight Allophycocyanin-Streptavidin (APC-SA), PerkinElmer) in assay diluent were dispensed into each well of the destination plate with the Multidrop Combi nL dispenser. After incubation at room temperature for 1 h, the destination plates were read on the PHERAstar FS plate reader (BMG Labtech) with donor excitation at 337 nm, donor emission at 615 nm and acceptor emission at 665 nm.

The signal was expressed as the ratio of RFUs in each well (RFU ratio = (665 nm/615 nm) × 10^4^). For ranking the binders, a double normalization was applied to obtain %DeltaF. DeltaF(%) = {(RFU [sample] − mean RFU [negative])/mean RFU [negative]} × 100. The RZ-score was calculated on the sample plate basis. For screening quality control, large-scale prepared positive control (A*02:01 with pp65) and a negative control (A*02:01 only) were added to designated wells in each sample plate. The acceptance of the screen was determined by the Z-factor calculated from the assay controls (Z-factor = 1 − {(3 s.d. [positive] – 3 s.d. [negative])/(mean [positive] − mean [negative])}). Sample plates with a Z-factor >0.4 were qualified for data processing.

### Untargeted MS and database search

See Supplementary Information for HLA-IP information. One-third of each sample was loaded into a 25 cm × 75 µm ID, 1.6 µm C18 IonOpticks Aurora Series column (IonOpticks, AUR2-25075C18A) on a Thermo UltiMate 3000 high-performance liquid chromatography (HPLC) system (Thermo Fisher Scientific) at a flow rate of 400 nl min^−1^. Peptides were separated with a 90-min gradient of 2% to 35% or 40% buffer B (98% ACN, 2% water and 0.1% FA) at a flow rate of 300 nl min^−1^. The gradient was further raised to 75% buffer B for 5 min and to 90% buffer B for 4 min at the same flow rate before final equilibration with 98% buffer A (98% water, 2% ACN and 0.1% FA) and 2% buffer B for 10 min at a flow rate of 400 nl min^−1^.

Peptide mass spectra were acquired using either an Orbitrap Fusion Lumos or an Orbitrap Eclipse Tribrid mass spectrometer (Thermo Fisher Scientific) with MS^1^ Orbitrap resolution of 240,000 and MS/MS fragmentation of the precursor ions by collision-induced dissociation (CID), followed by spectra acquisition at MS^2^ Orbitrap resolution of 15,000. All data-dependent acquisition (DDA) spectral raw files were searched in PEAKSOnline (Bioinformatics Solutions, PEAKS Online X build 1.7) against a UniProt-derived *Homo sapiens* proteome (downloaded on 3 October 2019) that contained appended concatenated sequences of the 47 most common mutations flanked by ~13-mer sequences on either end of each mutation with or without stretches of glycine and serine residue (GS) linkers along with sequences of BFP. Within PEAKSOnline, because HLA peptides are non-tryptic, the enzyme specificity was set as none; CID was selected as an activation method; and Orbitrap (Orbi-Orbi) was chosen as an instrument parameter. In-depth de novo assisted database search and quantification were performed with precursor mass error tolerance of 15 ppm, fragment mass error tolerance of 0.02 Da and missed cleavage allowance of 3. Carbamidomethylation (Cys+57.02) was set as a fixed modification, whereas deamidation (Asn+0.98 and Gln+0.98) and oxidation (Met+15.99) were set as variable post-translational modifications (PTMs), allowing a maximum of three variable PTMs per peptide. Additional report filters included peptide spectral match FDR of 1%, proteins −log_10_*P* ≥ 20 and de novo only amino acid residue average local confidence of 50%. For label-free analysis, a new group was created for each sample, and match between runs was performed with default parameters, except that retention time shift tolerance was set to 4 min and base sample was selected as ‘Average’. Output CSV files were exported and further analyzed in R.

### Targeted MS

See Supplementary Information for HLA-IP information. Absolute quantification (AQUA) synthetic heavy peptides (8–11-mer) (Elim Biopharmaceuticals) for all 47 mutation-derived neoantigens with TR-FRET RZ-score ≥5 or predicted NetMHC %Rank ≤2 (for a subset of mutations) were reconstituted in 30% ACN/0.1% formic acid (FA). DMSO was added for peptides that were not readily soluble in 30% ACN/0.1% FA. A working solution of 25 µM was made for each AQUA peptide from which allele-specific mastermix was made at 25 pmol per peptide. The peptides were reduced/alkylated and cleaned up with C18 cartridges on AssayMAP Bravo. After drying, the peptides were reconstituted in 0.1% FA/0.05% HFBA at 100 fmol per peptide. For each allele-specific assay, the intact modified mass was calculated for each peptide in that assay using TomahaqCompanion software^40^, which was then used to build an inclusion list MS method for a scouting run to get the retention time and mass-to-charge (*m*/*z*) of each target peptide. Then, 1 µl of each assay was injected into the IonOpticks C18 column and sprayed into the mass spectrometer for a 125-min run as described above, and the raw files were imported and analyzed in Skyline (64-bit, 19.1.0.193) to select appropriate charge for each peptide. A mass list table was built for each assay where a 4-min retention time window was created on both sides of the retention time for each target peptide, which was then imported into the Xcalibur instrument method application and saved as an allele-specific parallel reaction monitoring (PRM) method. For both Fusion Lumos and Eclipse instruments, MS^1^ was acquired at Orbitrap resolution of 240,000 with a maximum injection time of 50 ms, followed by a quadrupole isolation window of 1.2 *m*/*z*, CID fragmentation of parent ions, maximum injection time of 300 ms and MS^2^ acquisition at Orbitrap resolution of 60,000. For Eclipse acquisition, MS^1^ and MS^2^ AGC targets were set at 250% and 400%, respectively. One-third of each monoallelic sample was spiked with 100 fmol of corresponding AQUA mastermix and injected into the mass spectrometer using the same HPLC setup as described above. Raw PRM data were imported and analyzed in Skyline in an allele-specific manner. The ratios of the light peptides to their heavy counterparts across samples were exported as CSV files and further analyzed in R. For each neoepitope, background signal detected in the synthetic peptide-only analysis was subtracted from endogenous peptide signal before calculation of a final attomole amount. See Supplementary Information for methods relating to targeted MS quantification of full-length KRAS protein and neoepitopes.

### Ribo-Seq

Ribo-Seq was performed as previously described^41,42^. In brief, 8 million linker and no-linker HLA-A*02:01-engineered cells were lysed in polysome lysis buffer (20 mM Tris-HCl pH 7.4, 150 mM NaCl, 5 mM MgCl_2_, 1 mM DTT, 1% Triton X-100, 25 U ml^−1^ Turbo DNase and 0.1 mg ml^−1^ cycloheximide). The lysate volume equivalent to 30 μg of RNA was digested with 7.5 U of RNase I (LGC Biosearch Technologies) for 15 min at room temperature. Monosomes were purified using MicroSpin S-400 HR columns (Cytiva) according to the manufacturer’s instructions. The flow-throughs were mixed with TRI Reagent (Zymo Research), and the RNA was purified using the Direct-zol RNA Miniprep Kit (Zymo Research). Ribosome footprints were purified and size selected by electrophoresis in a 15% polyacrylamide TBE-Urea gel (Invitrogen), and footprints between 25 nucleotides (nt) and 32 nt were collected. rRNAs were depleted using the riboPOOL kit (siTOOLs Biotech) according to the manufacturer’s instructions. TruSeq Small RNA Library Preparation Kit (Illumina) was used for library preparation with modifications as previously described^41^. Libraries were purified by electrophoresis using 6% polyacrylamide TBE gels (Invitrogen). The quality of the libraries was confirmed on a D1000 ScreenTape (Agilent) on the 4200 TapeStation system (Agilent), and libraries were quantified using the Qubit dsDNA High-Sensitivity Assay (Invitrogen) on a Qubit 3.0 Fluorometer (Invitrogen). Sequencing was performed in a NovaSeq 6000 sequencer (Illumina) with a read length of 50 bp, single end, and depth of 100 million reads per sample.

Software used for the analysis of Ribo-Seq data was sourced from https://anaconda.org/ unless otherwise stated. Ribo-Seq reads were trimmed with Cutadapt (version 3.4_py38h4a8c8d9_1) with the following parameters: -j 8 -u 3 -u -5 -m 10 -a <adapter sequence file>. For RNA PCR primer sequences, see Supplementary Methods.

Next, reads aligning to rRNA and tRNA were removed using Bowtie (version 1.3.0_py38hcf49a77_2) with the following parameters: -p8 -v3–un. This procedure outputs a FASTQ file containing reads that map outside of rRNA and tRNA loci. These reads were aligned using STAR (version 2.7.10b) with two sets of parameters:

--outFilterType BySJout, outFilterMismatchNmax 2, outSAMtype BAM SortedByCoordinate, quantMode TranscriptomeSAM GeneCounts, outFilterMultimapNmax 1, outFilterMatchNmin 16, alignEndsType EndToEnd, runThreadN 16
--runMode alignReads--alignIntronMax 1--outFilterMismatchNmax 20--outFilterScoreMinOverLread 0.25--outFilterMatchNminOverLread 0.25--outSAMmode NoQS--outSAMtype BAM SortedByCoordinate--alignEndsType Extend5pOfReads12--outSAMattributes nM MD NH

SAMtools (version 1.13_h8c37831_0) was used for indexing and sorting the BAM files. De-duplication of the resulting BAM file was performed with Picard MarkDuplicates (version 2.25.7_hdfd78af_0). The de-duplicated BAM files were then indexed using SAMtools as mentioned previously. Lastly, BAM files were processed with three previously published Ribo-Seq ORF-calling and quality control programs: Price (version https://github.com/erhard-lab/gedi/releases/tag/Price_1.0.3b), RiboCode (version 1.2.11_pyh145b6a8_1) and RibORF (version https://github.com/zhejilab/RibORF/tree/master/RibORF.2.0). RiboCode accepts BAM files generated using the STAR alignment method (1), whereas Price and RibORF accept BAM files generated using the STAR alignment method (2).

### TCR discovery

A total of 376 predicted and MS-identified neoantigen-derived peptides were synthesized (GenScript), and each was added to six of 11 peptide pools such that each neoepitope (or group of similar neoepitopes) occupied a unique combination of six pools^5^. CD8^+^ T cells were isolated (STEMCELL Technologies) from healthy human donor leukopaks and expanded either on anti-CD3 coated plates (+anti-CD28/IL-2, BioLegend) or in the presence of matched donor-derived monocyte-derived dendritic cells^40^ and a pool of all 376 neoepitopes. At day 10–15, T cells were recovered, supplemented with one of the 11 neoepitope pools, incubated 8–14 h, enriched (Miltenyi Biotec) and then sorted using an anti-CD137 antibody (stained at 1/20: 5 µl of antibody was added to 100 µl of FACS buffer) (BioLegend). Sorted cells were then subjected either to immunoSEQ or pairSEQ (Adaptive Biotechnologies) to identify TCRB sequences displaying neoepitope-specific responsiveness and to associate TCRB with TCRA sequences in parallel, respectively. TCR sequences were encoded in pcDNA vectors as a single ORF, in the form of the full TCRB sequence followed by an RAKR motif and porcine teschovirus 2A cleavage peptide with the full TCRA sequence after in frame. TCR-encoding pcDNA vectors were then used as templates to generate TCR-encoding in vitro transcribed RNA (mMESSAGE mMACHINE, Thermo Fisher Scientific) for electroporation of primary human T cells.

### TCR reactivity assays

CD8^+^ cells were enriched from human peripheral blood mononuclear cells with EasySep Human CD8^+^ T Cell Isolation Kit (STEMCELL Technologies) and stimulated with 5 µg ml^−1^ Ultra-LEAF anti-human CD3 (BioLegend) and 2.5 µg ml^−1^ Ultra-LEAF anti-human CD28 (BioLegend). Cells were cultured in the presence of 20 ng ml^−1^ recombinant human IL-2 for 6 d. Human expanded CD8^+^ T cells were transfected with FLT3-p.D835Y-specific or PIK3CA-p.E545K-specific TCR RNA using a Lonza 4D-Nucleofector, P3 primary cell 4D-Nucleofector kit, program EO-115 (Lonza). RNA was purchased from TriLink BioTechnologies or in vitro transcribed. FLT3-p.D835Y-specific TCRs were co-cultured overnight with HLA-A*02:01-expressing T2 cells pulsed with YIMSDSNYV or HLA-A*02:01-expressing K562 cells transfected with a construct encoding the mutant or wild-type sequence. K562 cells were transfected using a Lonza 4D-Nucleofector, SF cell line 4D-Nucleofector kit, program FF-120 (Lonza). To determine specific cell lysis, an equal mixture of transfected HLA-A*02:01 + K562 cells and untransfected CellTrace Far Red (Thermo Fisher Scientific)-labeled HLA-A*02:01 + K562 cells was co-cultured overnight with T cells at a 2:1 effector-to-target ratio. Percent (%) specific cell lysis = (P_mock-transfected T cells_ − P_TCR-transfected T cells_)/(P_mock-transfected T cells_) × 100, where P is the proportion of transfected K562 targets relative to untransfected K562 cells, as measured by flow cytometry. CD137 expression on CD8^+^ T cells was assessed after an overnight co-culture with an anti-CD137 PE antibody (1/20: 5 µl of antibody was added to 100 µl of FACS buffer) (BioLegend).

PIK3CA-p.E545K-specific TCRs were co-cultured overnight with HLA-A*11:01-expressing K562s pulsed with STRDPLSEITK or transfected with a construct encoding the mutant or wild-type sequence. Equal mixtures of CellTrace Far Red-labeled HLA-A*11:01 + K562 cells were added to each well. T cell response to PIK3CA-presenting K562 cells was assessed as above.

### Resource availability

Further information and requests for resources and reagents should be directed to, and will be fulfilled by, the lead contact, Chris Rose (rose.christopher@gene.com). Plasmids generated in this study are the property of Genentech but can be made available under a material transfer agreement (MTA). Cell lines generated in this study are the property of Genentech but can be made available under an MTA. Recombinant HLA complexes generated in this study are the property of Genentech but can be made available under an MTA. MTAs can be requested at https://www.gene.com/scientists/mta.

### Reporting summary

Further information on research design is available in the Nature Portfolio Reporting Summary linked to this article.

## Online content

Any methods, additional references, Nature Portfolio reporting summaries, source data, extended data, supplementary information, acknowledgements, peer review information; details of author contributions and competing interests; and statements of data and code availability are available at 10.1038/s41587-023-01945-y.

### Supplementary information

Supplementary Information Supplementary Figs. 1–10 and Methods.

Reporting Summary

Supplementary Table 1 Data relating to cancer neoantigens and prevalent HLA alleles.

Supplementary Table 2 Data relating to the TR-FRET assay.

Supplementary Table 3 Data relating to MS analysis.

## Data Availability

Prevalence data for common cancer mutations (SNVs and indels) were obtained from the Cancer Hotspots database (http://cancerhotspots.org) and cross-referenced with TCGA data obtained from the cBioPortal for Cancer Genomics (http://cbioportal.org). Prevalence data for common HLA alleles were obtained by tabulating HLA types from the AFND (http://allelefrequencies.net) and from TCGA normal samples. All MS data have been deposited in the MASSIVE repository^43^ and are publicly available as of the date of publication under the identifier MSV000090323.
