# Supplementary material for: Systematic discovery of neoepitope–HLA pairs for neoantigens shared among patients and tumor types
Source: Nat Biotechnol. 2023 Oct 19;42(7):1107–17. doi: 10.1038/s41587-023-01945-y (PMC11251992; doi:10.1038/s41587-023-01945-y)
Supplement: Supplementary file 1 — Supplementary Figs. 1–10 and Methods. [file 41587_2023_1945_MOESM1_ESM.pdf]

nature biotechnology

Article https://doi.org/10.1038/s41587-023-01945-y

Systematic discovery of neoepitope–HLA pairs for neoantigens shared among patients and tumor types

In the format provided by the authors and unedited

Systematic discovery of neoepitope-HLA pairs for neoantigens shared among patients and tumor types

Gurung et al.

Supplementary Information

Included:
Supplementary Methods
10 Supplementary Figures

Supplementary Methods

Cell Lines

The HMy2.C1R, HOP62, NCIH2030, HuCCT1, SNU601, and SW527 cell lines were sourced from the Genentech cell back (gCell). These cell lines are authenticated by STR profiling, as previously described[1]. Cell lines used in the T Cell assays (T2 and K562) were sourced from ATCC.

Recombinant HLA and β2M Protein expression and purification

Recombinant HLA and β2M were over expressed in E. coli, purified from inclusion bodies, and stored in denaturing buffer (6M Guanidine HCl, 25 mM Tris pH 8) at -80°C as described previously[2]. Briefly, β2M and HLA biomass pellets were resuspended in lysis buffer (PBS+1% Triton X-114) at 5 mL/g and homogenized twice in a microfluidizer at 1000 bar. The homogenized suspension was spun at 30000 g for 20 min in an ultracentrifuge. The pellets were collected and washed with 500 ml of 0.5% Triton X-114 in PBS. Collected samples were then centrifuged at 30000 g for 20 min. The pellet was collected again and washed as described above. The purified inclusion bodies were dissolved in denaturing buffer (20 mM MES, pH 6.0, 6 M Guanidine) at a concentration of 10 ml/g and stirred at 4°C overnight. The dissolved pellet was centrifuged at 40000 g for 60 min and the supernatant was collected and filtered through a 0.22 mm filter. The concentration was determined by UV-vis at 280 nm using the protein's extinction coefficient. Samples were then snap-frozen and stored at -80°C prior to generation of complexes.

Peptides synthesis for *in vitro* binding assay

Peptides for the binding screen were synthesized by JPT Peptide Technologies GmbH (Germany) and purified to >70% purity by HPLC. Peptides were dissolved in ethylene glycol (Sigma) at 1 mg/mL and stored at -80°C in Matrix 1.0 mL 2D screw cap tubes (Thermo Scientific). UV-cleavable peptides were synthesized with 3-amino-3-(2-nitrophenyl)propionic acid by Elim Biopharm and purified by HPLC to >70% purity.

HLA peptide refold, biotinylation, and purification

Conditional HLA complexes were generated in a 5L refold reactions in refold buffer (100 mM Tris, pH 8.0, 400 mM L-Arginine, 2 mM EDTA) as described previously[3]. Briefly, the refold reaction consisted of the conditional HLA ligand peptide containing a nonnatural UV cleavable amino acid (0.01mM), oxidized and reduced glutathione (0.5mM and 4.0mM, respectively), recombinant HLA (0.03mg/ml) and β2M (0.01mg/ml). The refold mixture was stirred for 3-5 days at 4°C, filtered through a 0.22 µm filter, and concentrated and buffer exchanged by tangential flow filtration (TFF) (Millipore P2C010C01) into 25 mM Tris pH 7.5. The concentrated and refolded HLA complex was then biotinylated through the addition of BirA (1:50 (wt:wt) enzyme:HLA), 100 mM ATP and 10X reaction buffer (100 mM MgOAc, 0.5 mM biotin) for an incubation period of 2 hr at room temp. The sample was dialyzed and analyzed by LC/MS to quantify biotinylation. The biotinylated HLA complex was purified by anion exchange chromatography using a 1ml HiTrap Q HP column on an AKTA Avant FPLC. The column was equilibrated with 10 column volumes (CV) of 25 mM Tris-HCl pH 7.5 at a flow rate of 5 ml/min. The refolded peptide-HLA sample was loaded on the column at a 5 ml/min flow rate and eluted using 0-60% 2.5 mM TrisHCl, pH 7.5, 1 M NaCl gradient over 30 CV. Fractions across the eluted peak were run on SDS-PAGE, and fractions containing both β2M and HLA bands were pooled. Pooled fractions were buffer-exchanged into storage buffer (25 mM Tris HCl, pH 8.0, 150 mM NaCl). Protein concentration was determined by UV absorbance at 280 nm, and samples were snap-frozen and stored at -80°C. As previously described, QC for all conditional HLA complexes included SDS-PAGE, mass spec analysis, analytical SEC-MALS, and 2D LC-MS analysis of peptide exchange with a known binder[3].

Pan-HLA Antibody coupling and crosslinking

Pan-HLA Class I-specific antibody (clone W6/32) was coupled to Protein-A resin packed into AssayMAP Bravo compatible large capacity cartridges (PA-W 25 µL) (Agilent, Part number G5496-60018). The coupled antibodies were then crosslinked with 20 mM Dimethyl pimelimidate dihydrochloride (DMP) (Sigma-Aldrich, cat. D8388-250 MG) in 100 mM sodium borate crosslinking buffer at pH 9.0 immediately after the end of the coupling step. The impurities within the cartridges were washed away with simultaneous dispensing of 200 mM ethanolamine (Sigma-Aldrich, cat. E9508-100ML) pH 8.0 and deionized H_2O. The flow rates and other parameters of the affinity purification application within the AssayMAP Bravo software (VWorks) were used at the default settings. The antibody crosslinked Protein-

A cartridges were stored in rack filled with TBS/0.025% sodium azide, sealed with parafilm, and kept at 4°C.

Affinity purification of HLA-peptide complexes

Engineered monoallelic cell pellets (500 million cells/sample) were lysed at 4°C in 1% CHAPS (Roche Diagnostics, cat no. 10810126001) lysis buffer (pH 8.0) containing 20 mM TRIS, 150 mM NaCl, one tablet of cOmplete Protease Inhibitor Cocktail (Roche, cat. 4693159001) per 10 mL of the lysis buffer, and 0.2 mM phenylmethylsulfonyl fluoride (PMSF) (Sigma-Aldrich). The cell pellets were lysed with 2 mL of the lysis buffer by vortexing every 5 minutes for a total of 20 minutes at 4°C. The lysates were then transferred to LoBind tubes and centrifuged at 20,000 g at 4°C for 20 minutes. The supernatants were then carefully transferred to 0.45 µm polyethersulfone filter (Pall, cat. MCPM45C68). The samples were then centrifuged at 7000 g at 4°C for 30 minutes. The filtrate for each sample was carefully transferred to an AssayMAP Bravo compatible 96-well deep well plate making sure not to disturb any particulates that might have settled at the bottom of the conical tube. The deep well plate containing HLA-peptide complexes was transferred to the AssayMAP Bravo sample loading platform for automated dispensing of the samples through the W6/32 crosslinked Protein-A cartridges. The cartridges were primed and equilibrated with 20 mM Tris pH 8.0 and 150 mM NaCl in water. The sample impurities within the cartridges were washed away with automated dispensing of 20 mM Tris pH 8.0 and 400 mM NaCl in water followed by final wash with 20 mM Tris pH 8.0 in water. The antibody-bound HLA-peptide complexes were eluted with 0.1 M acetic acid in 0.1% trifluoroacetic acid (TFA). The flow rates and wash cycles were used at the default settings.

The eluates were transferred to ultra-low adsorption *ProteoSave* autosampler vials (AMR Incorporated cat. PSVial100) and dried in a speed vacuum. The dried samples were then reconstituted in 100 uL 20 mM HEPES pH 8.0, reduced with 5 mM Dithiothreitol (DTT) (Thermofisher, cat. A39255) in the dark at 65°C for 30 minutes, and alkylated with 15 mM Iodoacetamide (IAA) (Sigma-Aldrich, cat. I1149-5G) in the dark at RT for 30 minutes. The samples were then acidified with TFA to pH ~3.0, vortexed, and centrifuged at 14,000 g at RT for 5 minutes to pellet any debris. The samples were then carefully transferred to 96 well PCR, Full Skirt, PolyPro plate (Eppendorf, Part number 30129300) and loaded on the AssayMAP Bravo platform for final clean up before injection into the mass spectrometer. Four C18 cartridges (Agilent, Part number 5190-6532) were used per sample. The cartridges were primed with 80% acetonitrile (ACN) 0.1% TFA and equilibrated with 0.1% TFA. The samples were then loaded through the cartridges, washed with 0.1% TFA, and eluted with 30% ACN 0.1% TFA. After drying the samples in a speed vacuum, the samples

were reconstituted in 6 uL 0.1% formic acid (FA) 0.05% heptafluorobutyric acid (HFBA) (Thermo Fisher Scientific, cat. 25003).

Targeted Mass Spectrometry Quantification of Full Length KRAS Protein and Neoepitopes

For KRAS wild type (WT) and G12C/D/V mutant copy number presentation quantification in dox-inducible C1R A*11:01 KRAS full length (FL) cell lines and C1R A*11:01 47-neoantigen sample recombinant heavy isotope-coded peptide MHC (hipMHC)[4] monomers were made in-house for A*11:01 allele and KRAS WT/G12C/D/V (9 and 10 mer per target). These monomers were spiked at 1 pmol per 500 million cell lysate (KRAS FL samples) or 4.7 pmol per 500 million cell lysate (47-neoantigen sample) immediately before the pan HLA Class I affinity purification step. Similar to shared neoantigen samples, an inclusion method and a 125 minutes PRM method were developed for A*11:01 KRAS FL samples where only 8 AQUA peptides were in the hipMHC assay mix. The raw data were analyzed as described above with additional steps where on-column AQUA peptide concentration and input cell count were taken into consideration to calculate antigen copies per cell.

For absolute quantification of total KRAS WT and G12C/D/V proteins in dox-inducible C1R A*11:01 KRAS FL cell lines 20 million cells per sample were lysed in 1 mL 8M Urea lysis buffer 20 mM HEPES pH 8.0. 25 µg of yeast digest and 50 µg from each sample were spiked with 2.5 pmol KRAS quantification concatemer (QconCAT) polypeptide (Polyquant) generated by concatenation of heavy WT and select mutant RAS tryptic proteotypic peptides. Samples were reduced with 5 mM DTT in the dark at 56°C for 10 minutes with shaking and alkylated with 15 mM IAA in the dark at RT for 15 minutes. Urea concentration across control and sample tubes were dropped to ~2M with 20 mM HEPES pH 8.0 and were digested with 1 µg sequencing grade trypsin (Promega) overnight at 37°C in a nutator. Next day, trypsinization was quenched with 50% TFA and samples were cleaned up on C18 cartridges, dried, and reconstituted at 100 fmol/µL (yeast digest control) or 50 fmol/µL (samples) in 0.1% FA. The digested samples were run on Fusion Lumos mass spectrometer with a 65 minutes PRM method specific for RAS tryptic peptides present on QconCAT polypeptide. Data were analyzed on skyline and absolute quantification of each of the target KRAS peptides was calculated.

For the analysis of endogenous processing and presentation of KRAS neoepitopes, five G12X positive cancer cell lines including HOP62, NCIH2030, HuCCT1, SNU601, and SW527 that naturally express A*11:01 haplotype were grown at Analytical Biological Services (ABS) Inc at 500 million cells per pellet. All cells were grown in RPMI-1640, 10% FBS, and 2mM L-Glutamine. Each cell line was either left untreated or treated with recombinant Human interferon gamma (rhIFGg, R+D Biosystems #285-IF) at 100 U/mL for 48

hours. The cells were trypsinized, washed with cold PBS, snap frozen in liquid nitrogen, and transferred to -80 C. The cell pellets were lysed in 1% CHAPS lysis buffer as described earlier and HLA-peptide complexes were enriched with pan HLA Class I antibody on AssayMAP Bravo protein A cartridges. The recombinant heavy isotope-coded peptide MHC (hipMHC) monomers were made in-house for A*11:01 allele and KRAS G12C/D/V (9 and 10 mer per target). These monomers were spiked at 1 pmol per 500 million cell lysate immediately before the pan HLA Class I affinity purification step. The raw data were analyzed as described above with additional steps where on-column AQUA peptide concentration and input cell count were taken into consideration to calculate antigen copies per cell.

Ribso-Seq Primer Sequences

>RNA_PCR_Primer_(RP1)_part_#_15013198	TCGGACTGTAGAACTCTGAACGTGTAGATCTCGGTGGTCGCCGTATCATT
>RNA_PCR_Primer_Index_1_(RPI1)	TGGAATTCTCGGGTGCCAAGGAACTCCAGTCACATCACGATCTCGTATGCCGTCTTCTGCTTG
>RNA_PCR_Primer_Index_2_(RPI2)	TGGAATTCTCGGGTGCCAAGGAACTCCAGTCACCGATGTATCTCGTATGCCGTCTTCTGCTTG
>RNA_PCR_Primer_Index_3_(RPI3)	TGGAATTCTCGGGTGCCAAGGAACTCCAGTCACTTAGGCATCTCGTATGCCGTCTTCTGCTTG
>RNA_PCR_Primer_Index_4_(RPI4)	TGGAATTCTCGGGTGCCAAGGAACTCCAGTCACTGACCAATCTCGTATGCCGTCTTCTGCTTG
>RNA_PCR_Primer_Index_5_(RPI5)	TGGAATTCTCGGGTGCCAAGGAACTCCAGTCACACAGTGATCTCGTATGCCGTCTTCTGCTTG
>RNA_PCR_Primer_Index_6_(RPI6)	TGGAATTCTCGGGTGCCAAGGAACTCCAGTCACGCCAATATCTCGTATGCCGTCTTCTGCTTG
>RNA_PCR_Primer_Index_7_(RPI7)	TGGAATTCTCGGGTGCCAAGGAACTCCAGTCACCAGATCATCTCGTATGCCGTCTTCTGCTTG
>RNA_PCR_Primer_Index_8_(RPI8)	TGGAATTCTCGGGTGCCAAGGAACTCCAGTCACACTTGAATCTCGTATGCCGTCTTCTGCTTG
>RNA_PCR_Primer_Index_9_(RPI9)	TGGAATTCTCGGGTGCCAAGGAACTCCAGTCACGATCAGATCTCGTATGCCGTCTTCTGCTTG
>RNA_PCR_Primer_Index_10_(RPI10)	TGGAATTCTCGGGTGCCAAGGAACTCCAGTCACTAGCTTATCTCGTATGCCGTCTTCTGCTTG
>RNA_PCR_Primer_Index_11_(RPI11)	TGGAATTCTCGGGTGCCAAGGAACTCCAGTCACGGCTACATCTCGTATGCCGTCTTCTGCTTG
>RNA_PCR_Primer_Index_12_(RPI12)	TGGAATTCTCGGGTGCCAAGGAACTCCAGTCACCTTGTAATCTCGTATGCCGTCTTCTGCTTG

1. Yu, M. *et al.* A resource for cell line authentication, annotation and quality control. *Nature* 520, 307–311 (2015).

2. Jhunjhunwala, S., Hammer, C. & Delamarre, L. Antigen presentation in cancer: insights into tumour immunogenicity and immune evasion. *Nat Rev Cancer* 1–15 (2021) doi:10.1038/s41568-021-00339-z.

3. Darwish, M. *et al.* High-throughput identification of conditional MHCI ligands and scaled-up production of conditional MHCI complexes. *Protein Sci* 30, 1169–1183 (2021).

4. Stopfer, L. E., Mesfin, J. M., Joughin, B. A., Lauffenburger, D. A. & White, F. M. Multiplexed relative and absolute quantitative immunopeptidomics reveals MHC I repertoire alterations induced by CDK4/6 inhibition. *Nat Commun* 11, 2760 (2020).

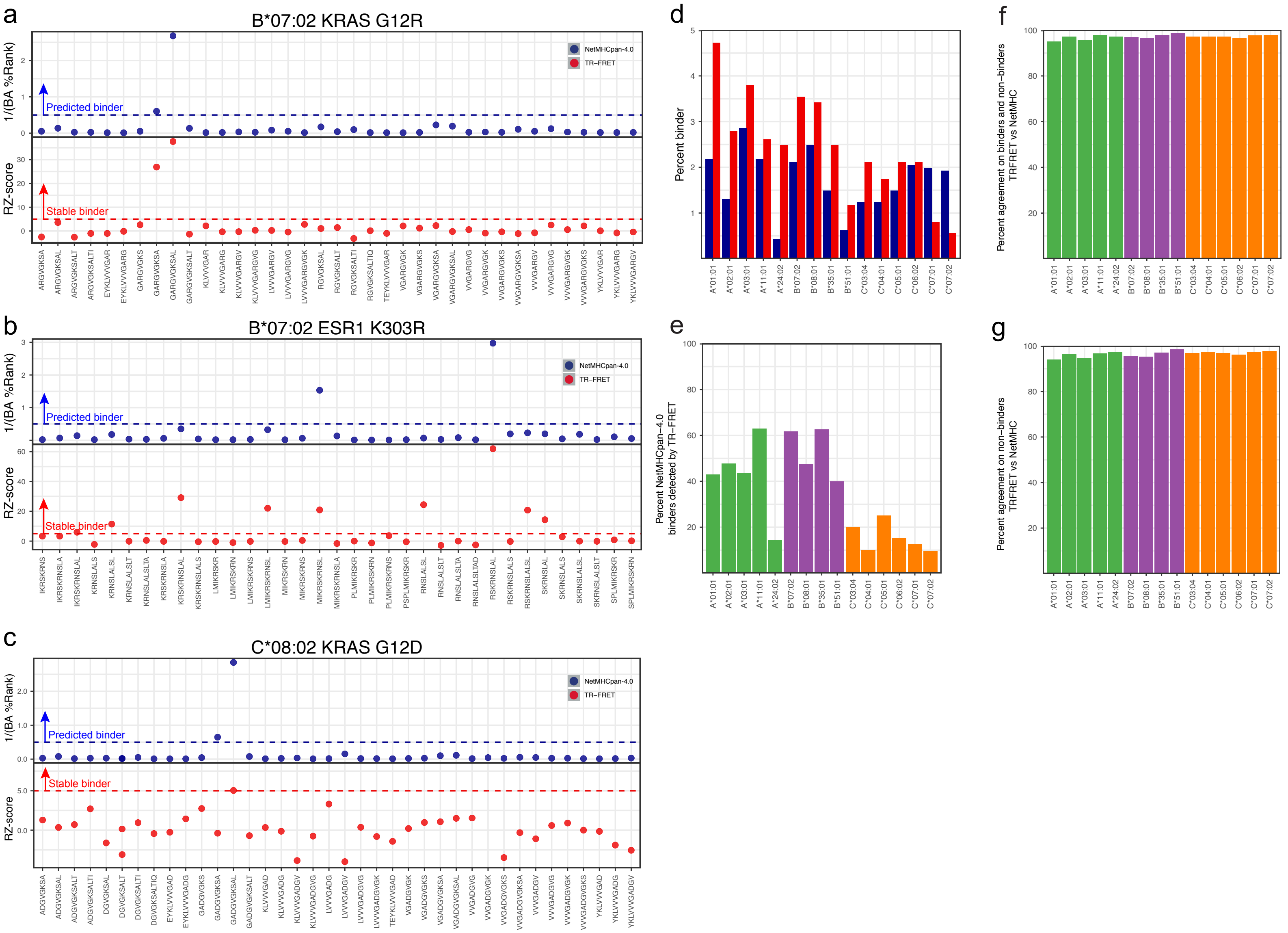

Supplementary Figure 1. Comparison of TR-FRET and NetMHC analysis. (a-c) TR-FRET Robust Z-score (red dots) and 1/NetMHC BA %Rank (blue dots) of all neoepitope-HLA combinations for B*07:02 and KRAS G12R (a), B*07:02 and ESR1 K303R (b), and C*08:02 and KRAS G12D (c). The blue and red lines represent the cutoff of stable binders for NetMHC and TR-FRET analysis, respectively. (d) Percent of neoepitope-HLA combinations that were determined to be stable binders by TR-FRET (red bars) and NetMHC (blue bars) analysis for each individual allele. (e) Percent neo-epitopes-HLA pairs found to be binders and non-binders by both NetMHC and TR-FRET across the individual alleles. (f) Percent neoepitopes-HLA pairs found to be binders by both NetMHC and TR-FRET across the individual alleles. (g) Percent neoepitopes-HLA pairs found to be non-binders by both NetMHC and TR-FRET across the individual alleles.

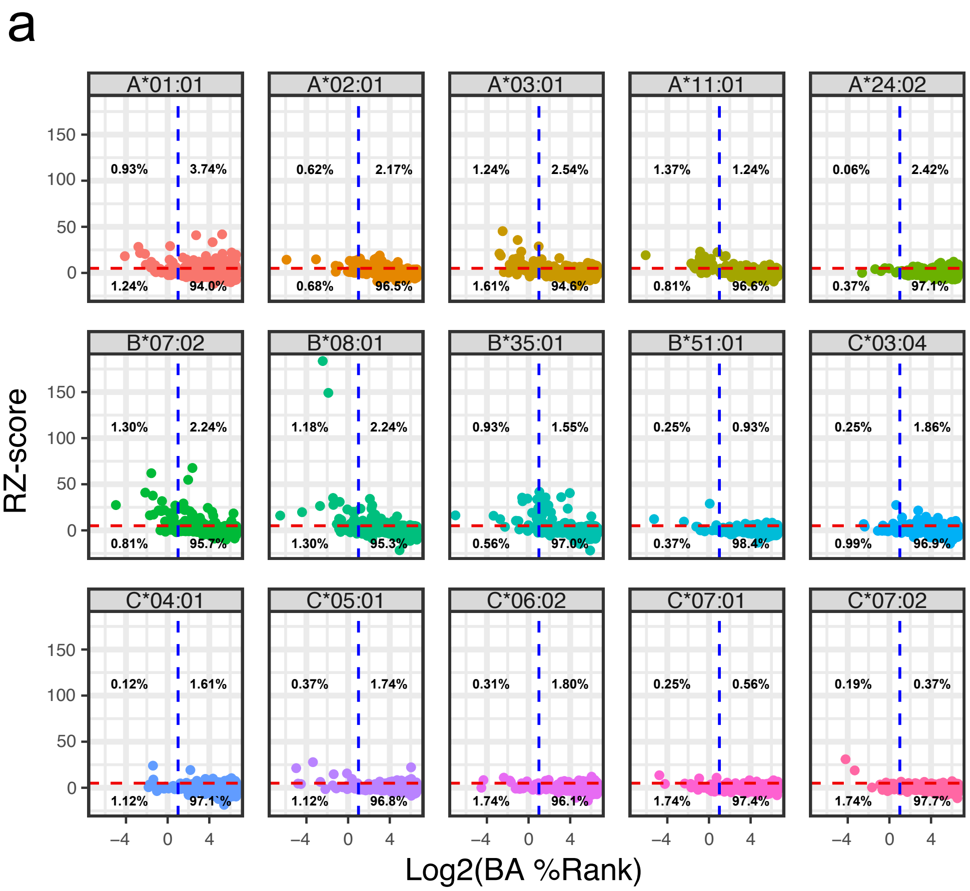

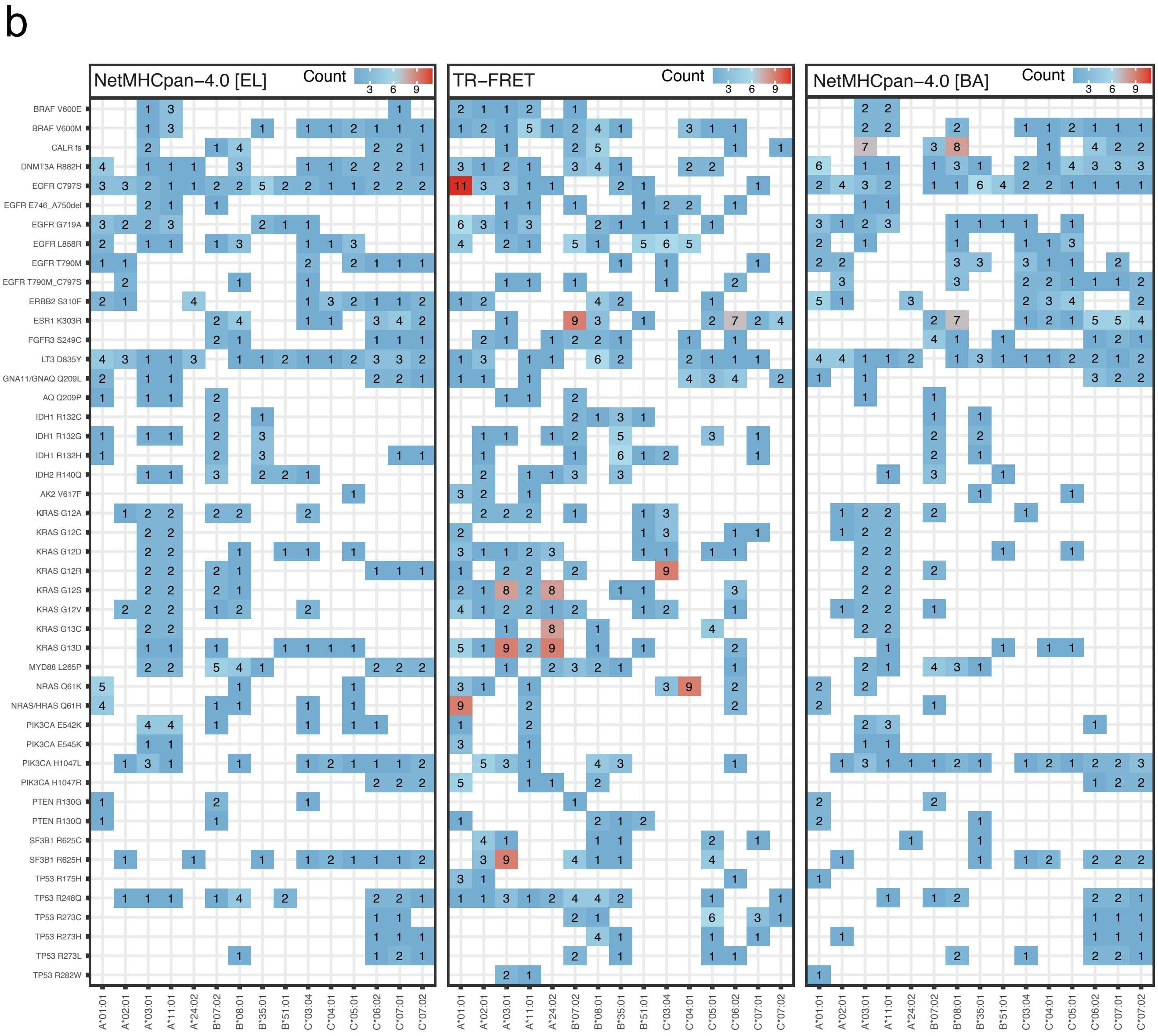

Supplementary Figure 2. Further comparison of Robust Z-score and NetMHC BA and EL %Rank across 15 alleles and neoepitopes. (a) Scatter plots of RZ-score and NetMHC BA %Rank for all peptide-HLA pairs assayed within the TR-FRET assay. The dashed red line represents the cutoff for stable binders as measured by TR-FRET, where values higher than the red line are considered stable binder. The dashed blue line represents the cutoff for binders based on NetMHC analysis, where values lower than the blue line are considered binders. (b) Heatmap representation of the number of stable binders (TR-FRET) as well as predicted binding (NetMHC BA) or presented peptides (NetMHC EL) for each neoantigen within a given HLA allele.

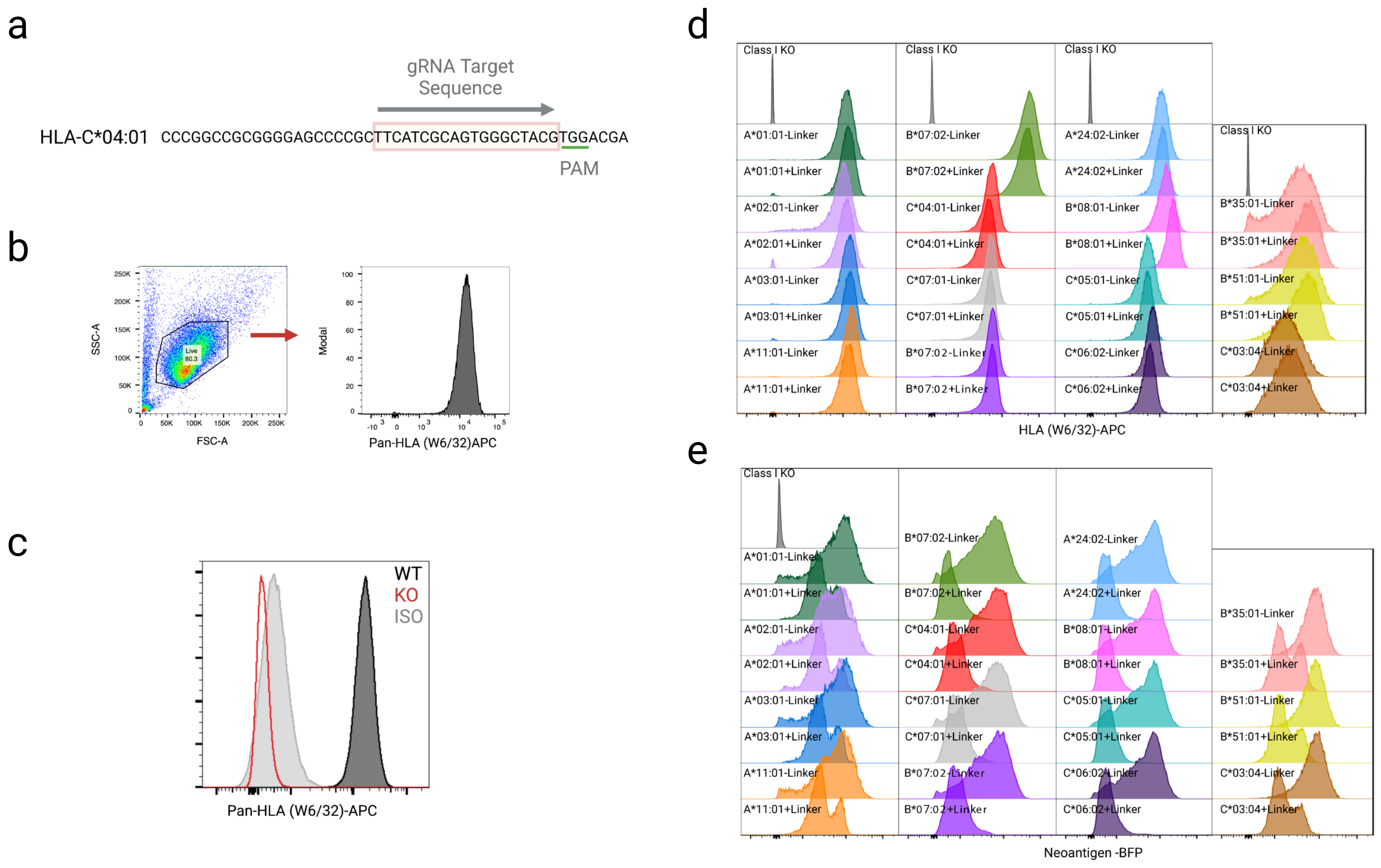

Supplementary Figure 3. Extended analysis of polyantigen expressing HLA class I monoallelic cell lines. (a) Guide RNA sequence and genomic context for CRISPR/Cas9 mediated gene disruption of the HLA-C locus in HMy2.C1R cells. (b) Gating strategy for live cell identification based on FSC-A/SSC-A profile. (c) Flow cytometric detection of pan-HLA-I expression in wild-type (WT) or HMy2.C1R HLA I-knockout (KO) cells using a pan-HLA-I detection antibody W6/32 (APC) or isotype (ISO) control. (d) Flow cytometric detection of pan-HLA-I expression of indicated monoallelic cell lines. (e) Flow cytometric detection of a transcriptionally linked TagBFP2 reporter gene in indicated polyantigen expressing monoallelic cell lines.

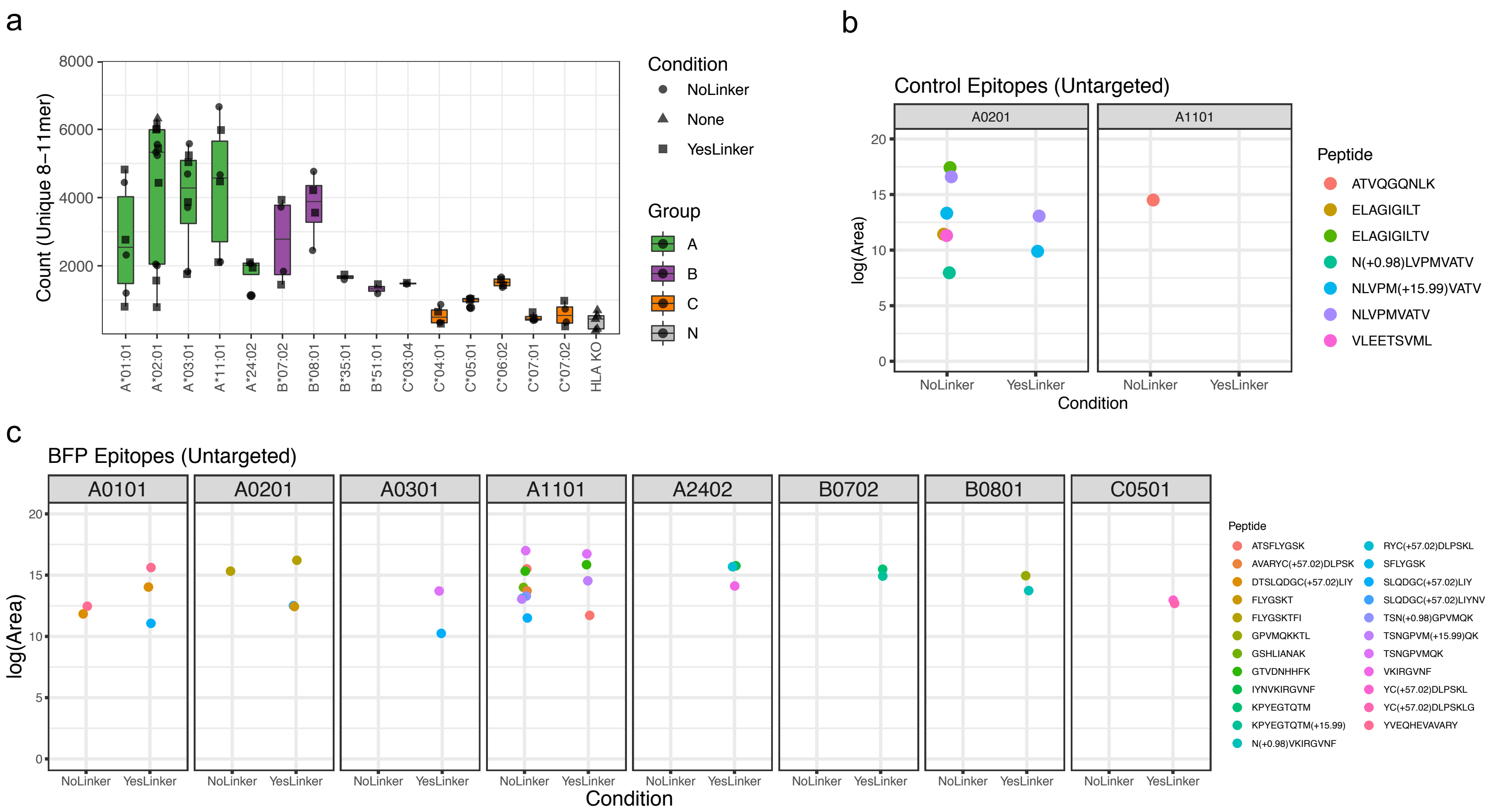

Supplementary Figure 4. Summary of peptides identified in untargeted immunopeptidomics. (a) Number of unique peptides (8-11 mer) identified in untargeted proteomics analysis stratified by allele and linker status of polyantigen construct. Each dot represents a seperate analysis begining with a different cell pellet [A*01:01 n=6, A*02:01 n=13, A*03:01 n=8, A*11:01 n=6, A*24:02 n=4, B*07:02 n=4, B*08:01 n=4, B*35:01 n=2, B*51:01 n=2, C*03:04 n=2, C*04:01 n=4, C*05:01 n=4, C*06:02 n=4, C*07:01 n=4, C*07:02 n=4, HLAKO n=5]. The box represents the inner quartile range, line represents the median value, and the whiskers represent the min and max value (excluding outliers). (b) Epitopes detected from control viral neoantigens located at the c-terminal end of the polyantigen cassette. Each dot represents the maximal area detected for the specific epitope. (c) Epitopes detected from BFP located after an IRES segment located on the c-terminal end of the polyantigen cassette. Each dot represents the maximal area

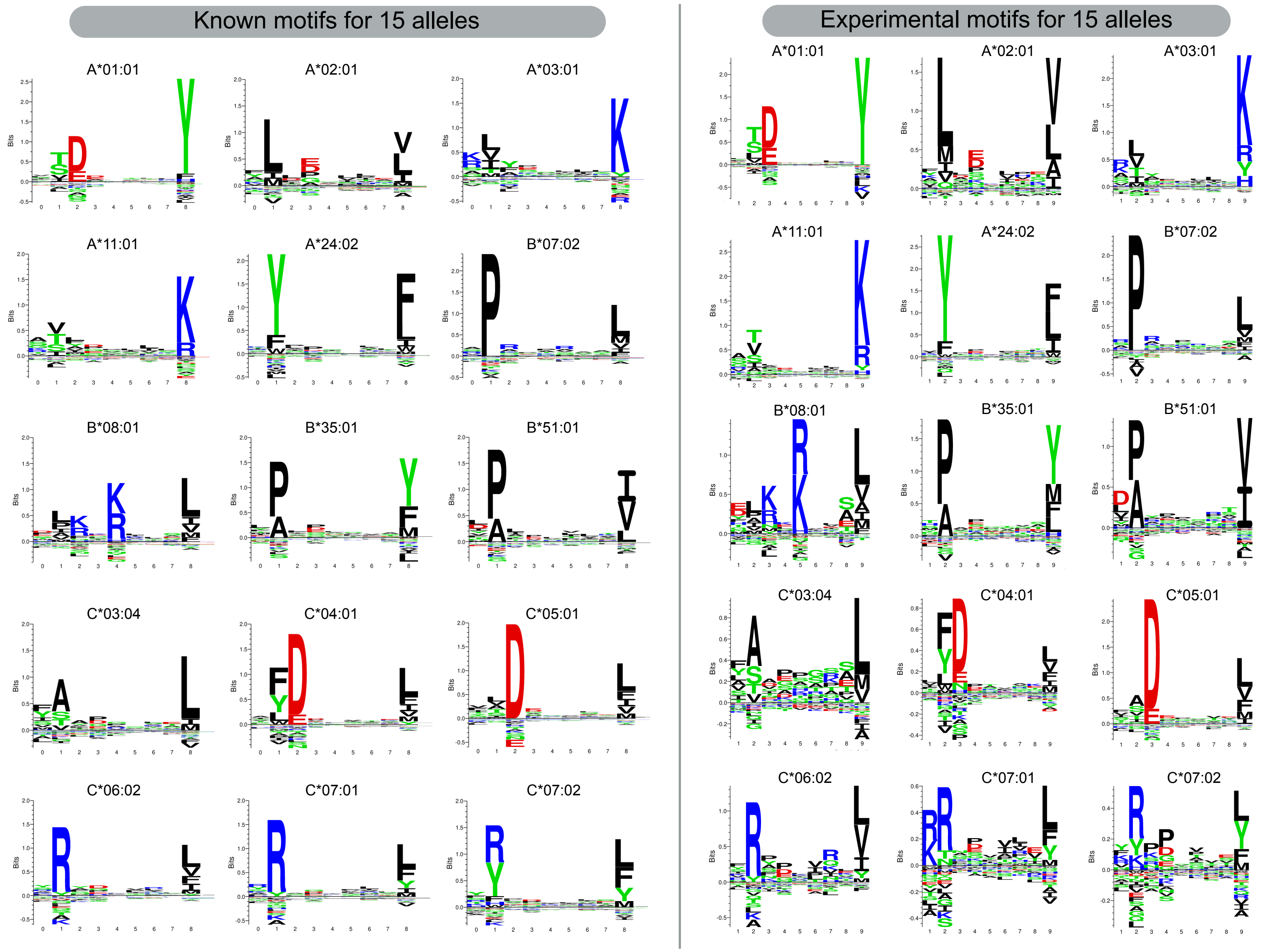

Supplementary Figure 5. Motif analysis of untargeted proteomic analysis. Motifs were generated with GibbsCluster 2.0 with two bins allowing for one bin of the dominant motif and a second bin for non-specific peptides.

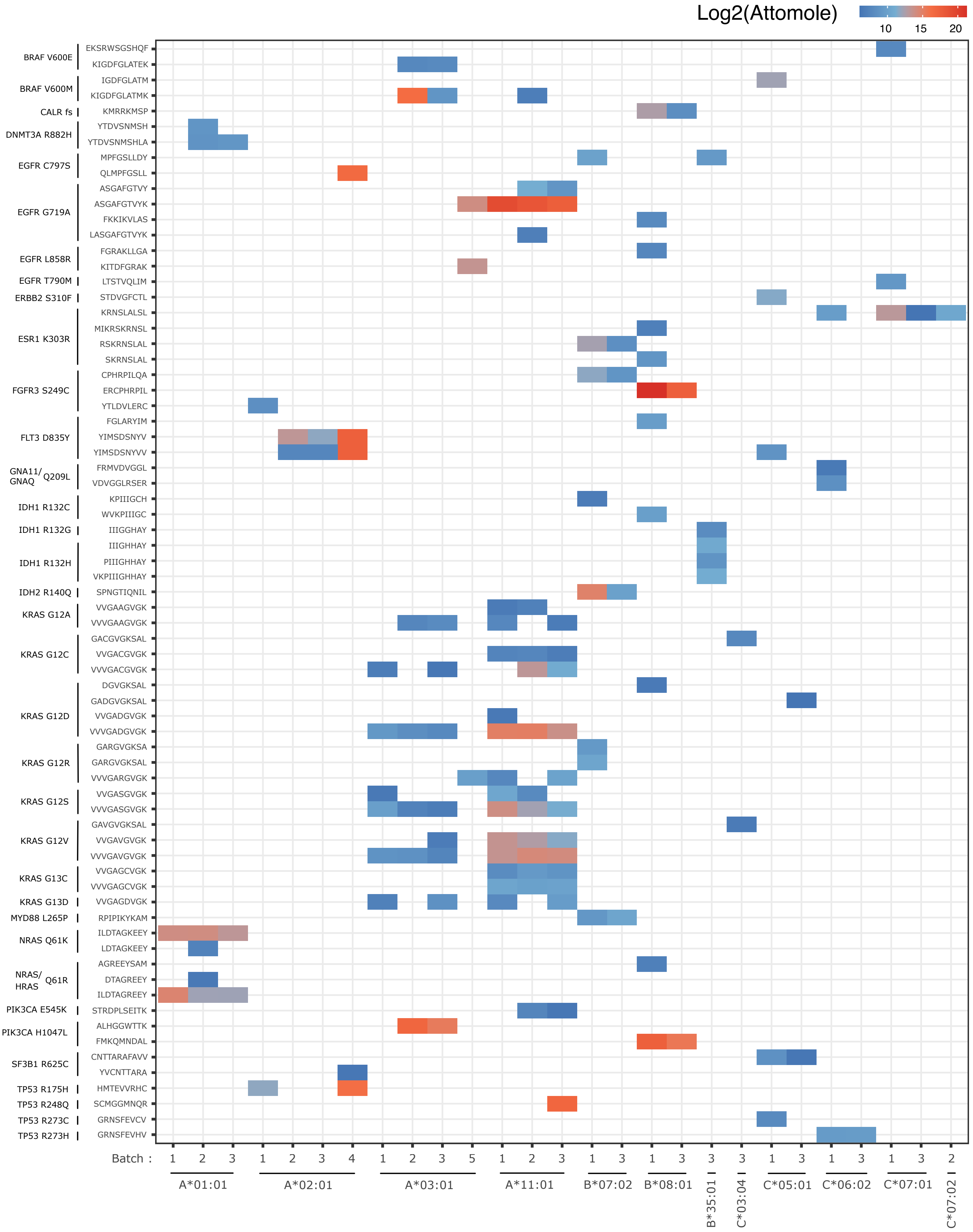

Supplementary Figure 6. Comparison of epitope presentation across analysis batches. For each allele, detection of epitopes displayed for each batch that contained analysis of monoallelic cell lines expressing that particular allele. Color represents the absolute abundance measured by the attomole amount detected on column for each neoepitope.

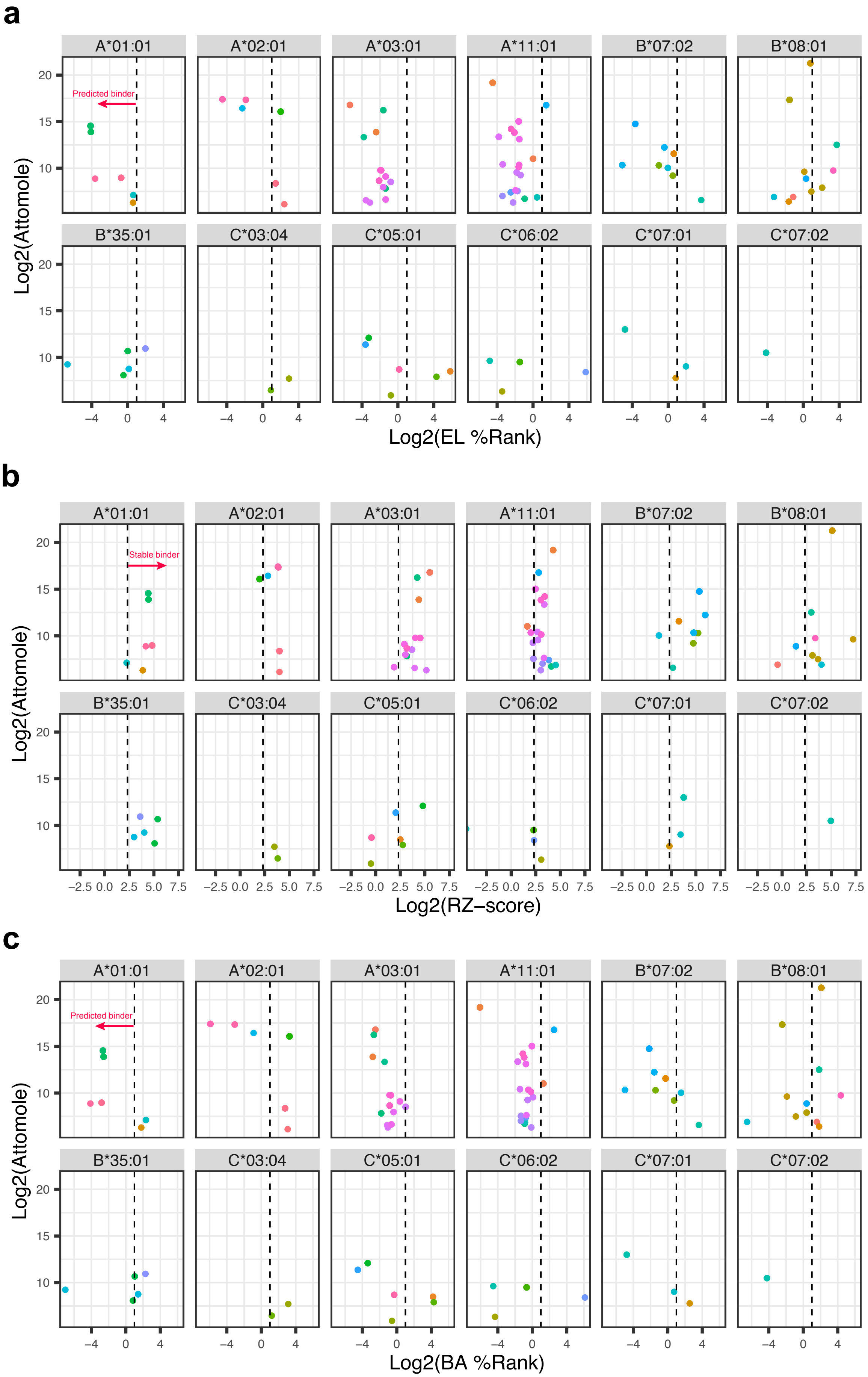

Supplementary Figure 7. Comparison of absolute peptide presentation and NetMHC BA %Rank, EL %Rank, or TR-FRET Robust Z-score. (a-c) Each dot represents a neoeptiope-HLA pair detected within the targeted proteomic analysis. If multiple neoepitope-HLA pairs were detected, the attomole value is the maximum value for that peptide-HLA pair across all analyses.

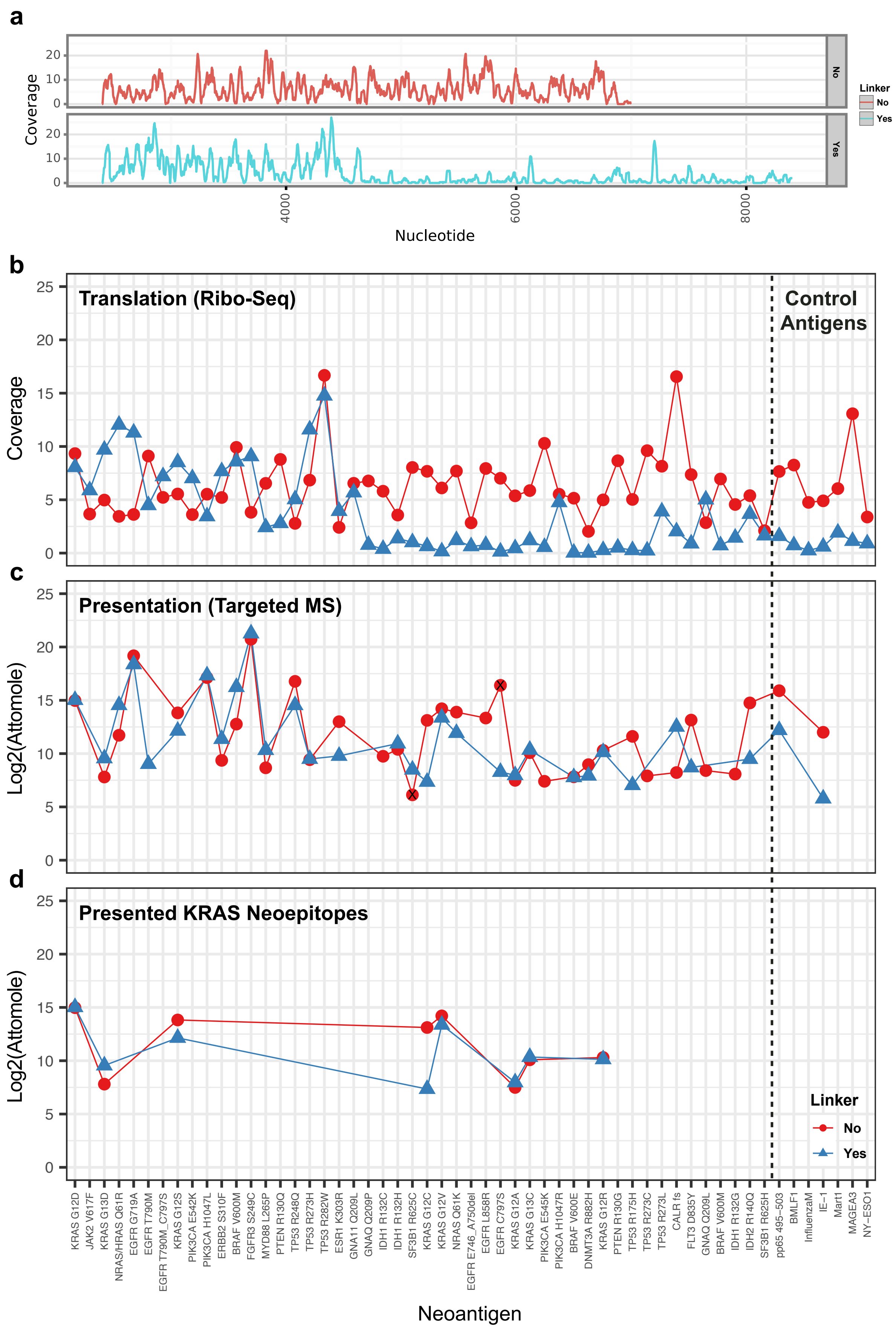

Supplementary Figure 8. Characterization of translation and presentation of linker a no-linker neoantigen cassettes. (a) Coverage as measured by Ribo-Seq as a function of the absolute number of nucleotides in the polyantigen cassette. (b) Results of Ribo-Seq analysis of A*02:01 cells containing either linker or no-linker polyantigen cassettes. Each point represents the coverage per neoantigen (n=3). (c) Maximum presentation of neoantigens detected across all targeted proteomic analyses irrespective of HLA allele. Circles with 'X' indicate a measurement from a monoallelic cell line containing a polyantigen cassette without control sequences. (d) Maximum presentation of KRAS neoepitopes detected within targeted proteomic analysis irrespective of HLA allele.

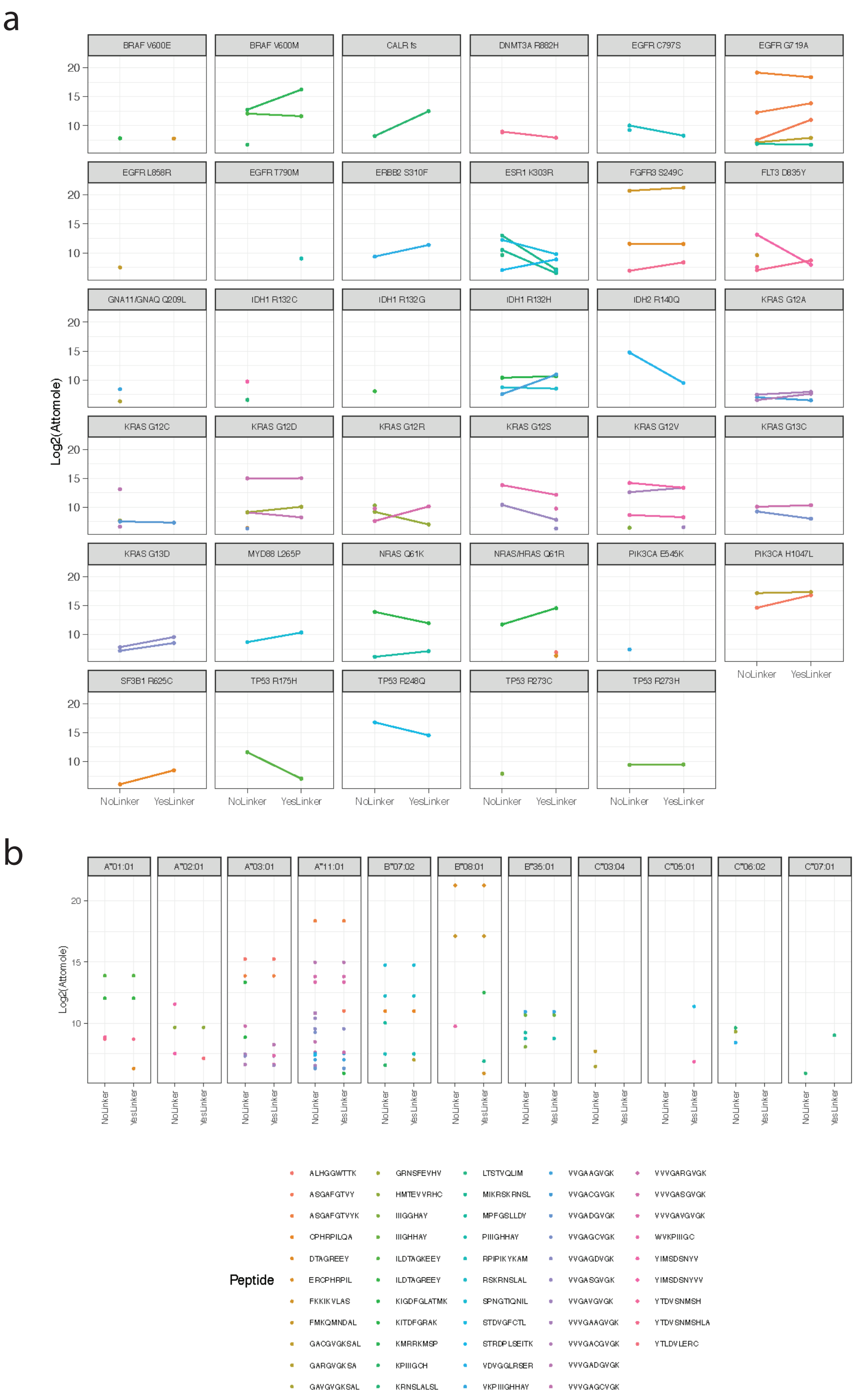

Supplementary Figure 9. Comparison epitope presentation for cell lines containing linker and no-linker construct. (a) Analysis of neoepitope presentation. Each line represents a specific epitope and the slope of the line demonstrates if expression was lower, higher, or similar between monoallelic cell lines containing linker and no-linker polyantigen cassettes. (b) For each allele the highest absolute amount of neoepitope is represented in cells containing linker or no-linker polyantigen cassettes

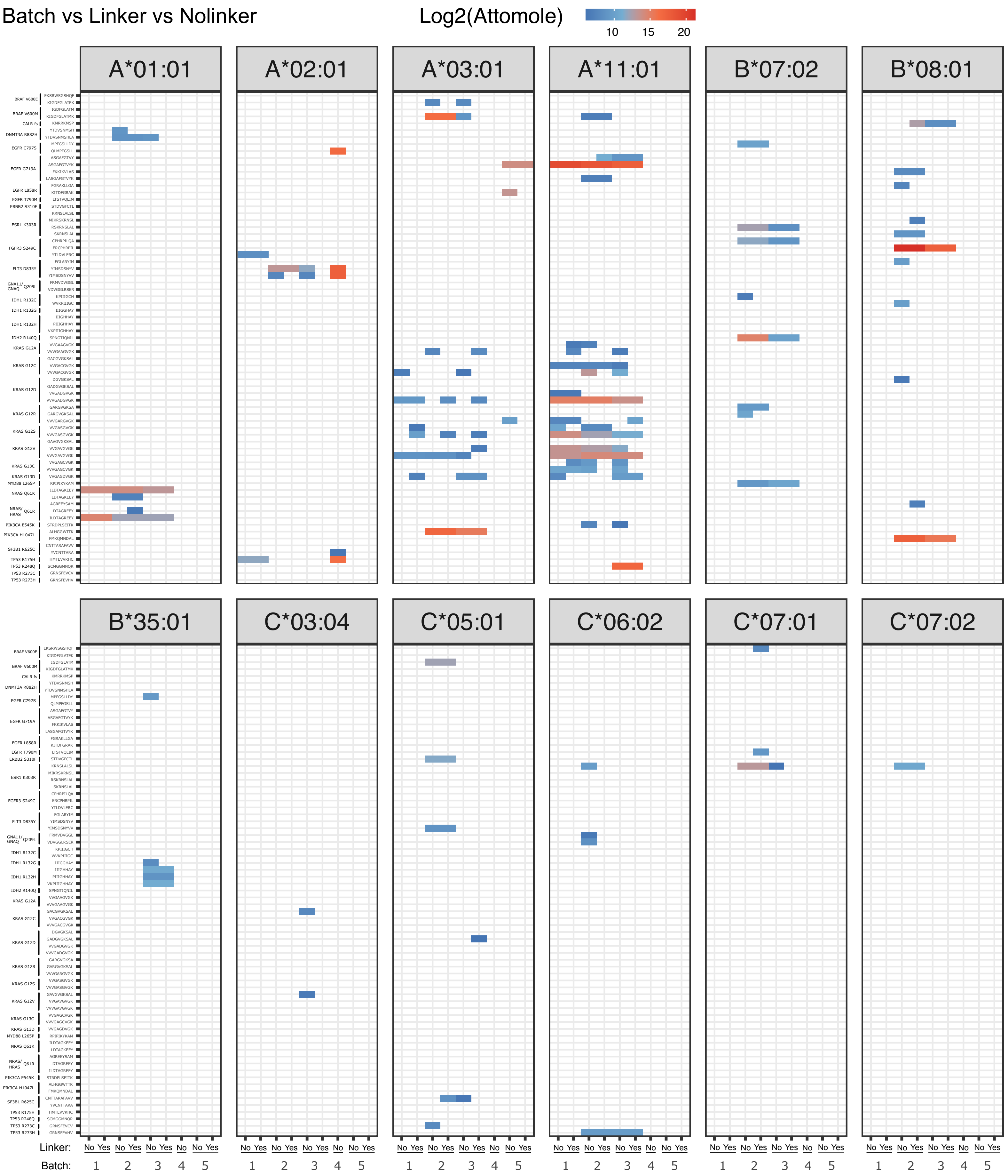

Supplementary Figure 10. Analysis of epitope presentation across analysis batches with consideration of presence of linker in construct. Each column represents a specific analysis batch for a cell line expressing a linker or no-linker polyantigen cassettes. Color represents the absolute abundance measured by the attomole amount detected on column for each neoepitope.
