# Supplementary material for: Systematic discovery of neoepitope–HLA pairs for neoantigens shared among patients and tumor types
Source: Nat Biotechnol. 2023 Oct 19;42(7):1107–17. doi: 10.1038/s41587-023-01945-y (PMC11251992; doi:10.1038/s41587-023-01945-y)
Supplement: Supplementary file 2 — Reporting Summary [file 41587_2023_1945_MOESM2_ESM.pdf]

nature portfolio

Corresponding author(s): Chris Rose, Benjamin Haley, Craig Blanchette

Last updated by author(s): Jul 25, 2023

Reporting Summary

Nature Portfolio wishes to improve the reproducibility of the work that we publish. This form provides structure for consistency and transparency in reporting. For further information on Nature Portfolio policies, see our Editorial Policies and the Editorial Policy Checklist.

Statistics

For all statistical analyses, confirm that the following items are present in the figure legend, table legend, main text, or Methods section.

n/a	Confirmed	
☐	☒	The exact sample size (*n*) for each experimental group/condition, given as a discrete number and unit of measurement
☒	☐	A statement on whether measurements were taken from distinct samples or whether the same sample was measured repeatedly
☐	☒	The statistical test(s) used AND whether they are one- or two-sided<br>*Only common tests should be described solely by name; describe more complex techniques in the Methods section.*
☒	☐	A description of all covariates tested
☒	☐	A description of any assumptions or corrections, such as tests of normality and adjustment for multiple comparisons
☐	☒	A full description of the statistical parameters including central tendency (e.g. means) or other basic estimates (e.g. regression coefficient) AND variation (e.g. standard deviation) or associated estimates of uncertainty (e.g. confidence intervals)
☐	☒	For null hypothesis testing, the test statistic (e.g. *F*, *t*, *r*) with confidence intervals, effect sizes, degrees of freedom and *P* value noted<br>*Give P values as exact values whenever suitable.*
☒	☐	For Bayesian analysis, information on the choice of priors and Markov chain Monte Carlo settings
☒	☐	For hierarchical and complex designs, identification of the appropriate level for tests and full reporting of outcomes
☒	☐	Estimates of effect sizes (e.g. Cohen's *d*, Pearson's *r*), indicating how they were calculated

Our web collection on statistics for biologists contains articles on many of the points above.

Software and code

Policy information about availability of computer code

Data collection	For flow cytometric data collection, experiments were performed on BD Celesta, BD Fortessa or BD Symphony machines using FACSDIVA v8/ v9 acquisition software.
Data analysis	Flow cytometry data was analyzed initially by FlowJo 2 v10.7/v10.8. Untargeted Mass Spectrometry was analyzed by PEAKS Online X build 1.7 (Bioinformatics Solutions Inc.). Targeted Mass spectrometry data were analyze with Skyline (64-bit, 19.1.0.193). TomahaqCompanion was used for retention time determination (https://github.com/CMRose3355/TomahaqCompanionProgram). Ribo-Seq data analysis utilized cutadapt (Version:3.4_py38h4a8c8d9_1), using bowtie (Version:1.3.0_py38hcf49a77_2), STAR (Version:2.7.10b), Samtools (Version:1.13_h8c37831_0), Picard MarkDuplicates (Version:2.25.7_hdfd78af_0), PRICE (Version:https://github.com/erhard-lab/gedi/ releases/tag/Price_1.0.3b), RiboCode(Version:1.2.11_pyh145b6a8_1), and RibORF (Version:https://github.com/zhejilab/RibORF/tree/master/RibORF.2.0).

For manuscripts utilizing custom algorithms or software that are central to the research but not yet described in published literature, software must be made available to editors and reviewers. We strongly encourage code deposition in a community repository (e.g. GitHub). See the Nature Portfolio guidelines for submitting code & software for further information.

Data

Policy information about availability of data

All manuscripts must include a data availability statement. This statement should provide the following information, where applicable:

- Accession codes, unique identifiers, or web links for publicly available datasets
- A description of any restrictions on data availability
- For clinical datasets or third party data, please ensure that the statement adheres to our policy

Data Availability: Prevalence data for common cancer mutations (SNVs and indels) were obtained from the Cancer Hotspots database (http://cancerhotspots.org) and cross-referenced with TCGA data obtained from the cBioPortal for Cancer Genomics (http://cbioportal.org). Prevalence data for common HLA alleles were obtained by tabulating HLA types from the Allele Frequency Net Database (AFND, http://allelefrequencies.net) and from TCGA normal samples. All mass spec data has been deposited in the MASSIVE repository (Wang et al., 2018) and are publicly available as of the date of publication under the identifier MSV000090323.

Human research participants

Policy information about studies involving human research participants and Sex and Gender in Research.

Reporting on sex and gender	N/A
Population characteristics	N/A
Recruitment	N/A
Ethics oversight	N/A

Note that full information on the approval of the study protocol must also be provided in the manuscript.

Field-specific reporting

Please select the one below that is the best fit for your research. If you are not sure, read the appropriate sections before making your selection.

☒ Life sciences ☐ Behavioural & social sciences ☐ Ecological, evolutionary & environmental sciences

For a reference copy of the document with all sections, see nature.com/documents/nr-reporting-summary-flat.pdf

Life sciences study design

All studies must disclose on these points even when the disclosure is negative.

Sample size	Sample size for each experiment indicated in the figure legend, methods or associated text. Sample sizes were determined by the number of replicates needed to provide confidence in the data or what was feasibly possible within the limitations of resources.
Data exclusions	A technical replicate may be missing due to technical problems/sample loss during data collection.
Replication	All comparative studies were performed a minimum of 2 times with representative experiments depicted. Biological and technical replicates for each experiment indicated in figure legend, methods or associated text.
Randomization	Randomization was applied when appropriate (e.g., order in which MS runs were performed). However, for most experiments randomization does not apply.
Blinding	Blinding was not possible as the researcher performing the experiment needed to know the components of the sample in order to perform sample preparation and analysis.

Reporting for specific materials, systems and methods

We require information from authors about some types of materials, experimental systems and methods used in many studies. Here, indicate whether each material, system or method listed is relevant to your study. If you are not sure if a list item applies to your research, read the appropriate section before selecting a response.

Materials & experimental systems

n/a	Involved in the study
☐	☒ Antibodies
☐	☒ Eukaryotic cell lines
☒	☐ Palaeontology and archaeology
☒	☐ Animals and other organisms
☒	☐ Clinical data
☒	☐ Dual use research of concern

Methods

n/a	Involved in the study
☒	☐ ChIP-seq
☐	☒ Flow cytometry
☒	☐ MRI-based neuroimaging

Antibodies

Antibodies used

Antibody clone, company and staining protocol listed in methods antibodies.

Validation

-W6/332-
Anti-human HLA-A,B,C mouse monoclonal;
Biolegend 311434 (biotin);311410 (APC):

Validated by Biolegend and:
Darrow TL, et al. 1989. J. Immunol. 142:3329.
Stern P, et al. 1987. J. Immunol. 138:1088.
Tran TM, et al. 2001. Immunogenetics 53:440.
Barbatis C, et al. 1981. Gut 22:985.
Ayyoub M, et al. 2004. Cancer Immunity 4:7.
DeFelice M, et al. 1990. Cell. Immunol. 126:420.
Fayen J, et al. 1998. Int. Immunol. 10:1347.
Turco MC, et al. 1988. J. Immunol. 141:2275.
Geppert TD, et al. 1989. J. Immunol. 142:3763.
Wooden SL, et al. 2005. J. Immunol. 175:1383.
Nagano M, et al. 2007. Blood 110:151.
McLoughlin RM,et al.2008. J. Immunol. 181:1323.
https://www.biolegend.com/en-us/products/biotin-anti-human-hla-a-b-c-antibody-12535

-Anti-CD137-
Anti-CD137 PE: Biolegend cat# 309804, Clone 4B4-1
Validation by Biolegend and:
1. Liu T, et al. 2021. Gut. 70:1965.
2. Shitaoka K, et al. 2018. Cancer Immunol Res. 6:378.
3. Arce Vargas F et al. 2018. Cancer cell. 33(4):649-663 .
4. Yamaguchi S, et al. 2021. Eur J Immunol. 51:2306.
5. Lamichhane R, et al. 2020. Eur J Immunol. 50:178.
6. Goncharov MM, et al. 2022. Elife.
7. Shin JJ, et al. 2022. J Clin Immunol.
8. Jutz S, et al. 2016. J Immunol Methods. 430:10-20.
9. Pavlicek D, et al. 2017. Immun Ageing. 14:22.
10. Gordon-Alonso M,et al. 2017. Nat. Commun. 10.1038/s41467-017-00925-6.
11. Rajamanickam V, et al. 2021. Cancer Immunol Res. 9:602.
12. Jung JH, et al. 2021. Nat Commun. 12:4043.

Anti-CD8 FITC: Biolegend cat# 300906, Clone HIT8a
Validation by Biolegend and:
1. Zhang H, et al. 2021. Front Immunol. 12:644520.
2. Bilich T, et al. 2021. Cancer Discov. 11:1982.
3. Galindo-Albarrán A, et al. 2016. Cell Rep. 17:2151-2160.
4. Thaker YR, et al. 2022. Front Oncol. 12:884196.
5. Sun L, et al. 2020. J Diabetes Res. 2020:2583257.
6. Zhou Y, et al. 2017. Front Cell Infect Microbiol. 7:457.
7. Mender I, et al. 2020. Cancer Cell. 38(3):400-411.e6.
8. Warmuth S, et al. 2022. Oncoimmunology. 10:2004661.
9. Shen X, et al. 2021. Front Immunol. 12:710750.
10. Bratke K, et al. 2017. Clin Exp Allergy. . 10.1111/cea.13017.
11. Durning S, et al. 2016. J Immunol. 197: 3076 - 3085.
12. Chulpanova DS, et al. 2021. Biology (Basel). 10

Biotin anti-human CD137 (4-1BB) Antibody, Biolegend, cat# 309806, clone 4B4-1
Validation by Biolegend and:
1. Garni-Wagner B, et al. 1996. Cell. Immunol. 169:91.
2. Salih HR, et al. 2000. J. Immunol. 165:2903.
3. Kienzle G, et al. 2000. Int. Immunol. 12:73.
4. Langstein J, et al. 1998. J. Immunol. 160:2488.

Eukaryotic cell lines

Policy information about cell lines and Sex and Gender in Research

Cell line source(s)	Cell lines sourced from the Genentech cell bank (gCell):<br>HMy2.C1R<br>HOP62<br>NCIH2030<br>HuCCT1<br>SNU601<br>SW527<br>HEK293T<br><br>Cell lines sourced from ATCC: (CONFIRM WITH ADAPTIVE)<br>T2<br>K562
Authentication	gCell sourced cell lines authenticated by STR profiling as described doi.org/10.1038/nature14397. T2 cells and K562 cells were used from ATCC without further authentication.
Mycoplasma contamination	Cells tested negative for mycoplasma contamination were maintained under mycoplasma-free conditions.
Commonly misidentified lines<br>(See ICLAC register)	No commonly misidentified lines were used in these studies.

Flow Cytometry

Plots

Confirm that:

☒ The axis labels state the marker and fluorochrome used (e.g. CD4-FITC).

☒ The axis scales are clearly visible. Include numbers along axes only for bottom left plot of group (a 'group' is an analysis of identical markers).

☒ All plots are contour plots with outliers or pseudocolor plots.

☒ A numerical value for number of cells or percentage (with statistics) is provided.

Methodology

Sample preparation	w6/32 Biolegend 311410 (APC) Pre-wash cells 1 X with staining buffer. Stain: 5ul antibody in 100ul staining buffer. Incubation 20-30min @ 4deg. Wash: 2 X with staining buffer.
Instrument	BD Celesta, BD Fortessa or BD Symphony
Software	FACSDIVA v8/v9 was utilized as acquisition software and FlowJo 2 v10.7/v10.8 was used for subsequent analysis
Cell population abundance	Live cell were defined by FSC-A/SSC-A profiles as indicated Supplementary Fig. 3b.
Gating strategy	Gating strategy indicated in Supplementary Fig. 3b. Live cell populations were gated using fsc and ssc profiles. HLA positive cells were stained with pan-HLA (w6/32) antibody staining and evaluated for APC signal. Polyneoantigen expressing cells specifically were identified by detection of the transcriptionally linked mTagBFP2.

☒ Tick this box to confirm that a figure exemplifying the gating strategy is provided in the Supplementary Information.
